# Heat-related mortality in Mexico: A multi-scale spatial analysis of extreme heat effects and municipality-level vulnerability

**DOI:** 10.1016/j.envint.2024.109231

**Published:** 2024-12-20

**Authors:** Lara Schwarz, Chen Chen, Javier Emmanuel Castillo Quiñones, L.C. Aguilar-Dodier, Kristen Hansen, Jaime Reyes Sanchez, David J.X. González, Gordon McCord, Tarik Benmarhnia

**Affiliations:** a School of Public Health, San Diego State University, San Diego, CA, USA; b Herbert Wertheim School of Public Health and Longevity Science, University of California San Diego, La Jolla, CA, USA; c Division of Environmental Health Sciences, School of Public Health, University of California Berkeley, Berkeley, CA, USA; d Scripps Institution of Oceanography, University of California San Diego, La Jolla, CA, USA; e Facultad de Ciencias Químicas e Ingeniería, Universidad Autónoma de Baja California, Tijuana, México; f Axle Research and Technology, Rockville, MD, USA; g Department of Population and Public Health Sciences, Keck School of Medicine, University of Southern California, CA, USA; h School of Global Policy and Strategy, University of California, San Diego, La Jolla, CA, USA; i Irset Institut de Recherche en Santé, Environnement et Travail, UMR-S 1085, Inserm, University of Rennes, EHESP, Rennes, France

**Keywords:** Extreme heat, Social vulnerability, Mexico, Spatial analysis

## Abstract

Understanding effects of extreme heat across diverse settings is critical as social determinants play an important role in modifying heat-related risks. We apply a multi-scale analysis to understand spatial variation in the effects of heat across Mexico and explore factors that are explaining heterogeneity. Daily all-cause mortality was collected from the Mexican Secretary of Health and municipality-specific extreme heat events were estimated using population-weighted temperatures from 1998 to 2019 using Daymet and WorldPop datasets. We analyzed the association between single-day extreme heat events defined at the 99th percentile of the same-day maximum temperature and mortality, and seven heat threshold metrics based on relative and absolute scales were considered as sensitivity analyses. A time-stratified case-crossover was applied to evaluate heat impacts across 32 states in Mexico. A within-community matched design with Bayesian Hierarchical model explored effects across 2456 municipalities. A random-effects meta-regression was applied to understand which municipality-level socio-demographic characteristics such as education, age and housing predicted observed spatial heterogeneity. Extreme heat increased the odds of mortality overall, and this was consistent across extreme heat thresholds. At the state level, extreme heat events showed highest impact on mortality in Tabasco [OR: 1.23, 95% CI: 1.17, 1.30]. The municipality-level spatial analysis showed substantial differences across regions with highest effects observed along the eastern, southwestern and Sonora coasts. Municipalities with older populations, higher marginalization, lower education, and poorer housing conditions were more vulnerable to heat effects. Understanding the differential risks of extreme heat events at varying scales is important to prioritize at-risk populations in action plans and policies to reduce their burden.

## 1. Introduction

Climate change constitutes a pressing global health and environmental justice issue. Countries and communities that contribute the least to anthropogenic greenhouse gas emissions experience a disproportionate burden, particularly from extreme heat (Vicedo-Cabrera et al. 2021). A far greater number of people vulnerable to poverty will be exposed to climate-related risks as the globe continues to warm (Masson-Delmotte et al. 2018). Generating evidence on the health effects of extreme heat and identifying what communities are more vulnerable can be used to allocate resources to vulnerable areas. This has been shown to maximize the potential effectiveness of actions in decreasing health burden and promoting health equity (Benmarhnia et al. 2016). Yet, epidemiological evidence regarding health impacts of extreme heat is predominantly produced in high-income countries (Campbell et al. 2018; Ebi et al. 2021; Green et al. 2019).

There is comparatively little epidemiological evidence regarding the impacts of extreme heat on health in Mexico, and few prior studies have explored these associations. Guerrero Compeán (2013) studied the relationship between extreme temperature and mortality rates, concluding that extreme heat drove increases in mortality, with higher vulnerability in rural areas (Guerrero Compeán, 2013). Austria and Bandala (2016) analyzed maximum temperature trends and the corresponding heat wave thresholds in the northwestern city of Mexicali, Mexico, and found an upward trend in heat waves in the past decades, identifying their presence mainly during July and August (Martínez Austria and Bandala 2016). This trend is also supported by the number of admissions and casualties registered in hospitals in the city of Mexicali (Cueto and García 2024). Work by Cohen and Dechezleprêtre (2022) explored the impact of temperature on mortality in Mexico and showed that 3.8 percent of deaths in the country are caused by suboptimal temperature, but found that the majority are driven by cold temperatures (Cohen and Dechezleprêtre 2022). Variations in the observed effects may be driven by regional-level differences in the population composition and temperature distributions. Measures of extreme heat events at a local scale have been shown to differ across regions, which shows the importance in evaluating different metrics for extreme heat events (McElroy et al. 2019; Tong and Kan 2011).

The health effects of heat will not impact everyone equally and some communities will be diproportionately affected. Many factors may contribute to increased risk, including housing characteristics, linguistic isolation, or increased physiological vulnerability due to other social and/or biological stressors (Gronlund 2014; Jones et al. 2021). Populations with a lower socio-economic status (SES) and ethnic minority groups are more likely to live in warmer neighborhoods that have high settlement density, sparse vegetation, and no open space which are aspects of the built environment that can increase heat stress (Harlan et al. 2006; Hsu et al. 2021). Poorer physical health, less health insurance coverage, and higher levels of crime in the neighborhoods can all contribute to increased vulnerability (Gronlund 2014). Weathering or accelerated biological aging due to psychosocial stress disproportionally affect racially minoritized populations and also increase vulnerability (Forrester et al. 2019). There is an urgent need for policy-relevant evidence to inform interventions to protect populations in the most vulnerable regions facing heat extremes.

In the current study, we expand on prior work and evaluate the effects of extreme heat on mortality risk at different geographical scales considering extreme heat thresholds based on local climate. As previous research in Mexico evaluates heat effects that country level, we conduct this analysis for comparison. We study state and municipality-level effects to evaluate the importance of going beyond average measures to consider finer-scale estimates in understanding the effects of heat across space (Schwarz et al. 2021). We then examine heterogeneity in these effects at the municipality-level using a spatial model. First, we estimate a spatially resolved measure of extreme heat using a local temperature distribution at the municipality level. Second, we explore the effects of heat across the country and between Mexican states using a time-stratified case-crossover design. Third, we examine the effects of extreme heat events on mortality at the municipality level using a within-community matched design and spatial Bayesian Hierarchical model (BHM). Lastly, we explore what municipality-specific socio-demographic characteristics are important predictors of the observed differences in the municipality-level effects of extreme heat on mortality. Understanding specific factors that are associated with increased risk among diverse communities can be used to inform targeted public health interventions to reduce the burden from heat and related health disparities.

## 2. Methods

### 2.1. Data sources

Mexico is composed of 2456 municipalities in 32 federal entities (31 states and Mexico City). All-cause mortality data were extracted from death certificates from the Mexican Secretary of Health and aggregated as daily counts from 1998, the earliest year of available data, to 2020 for all 2456 municipalities included in the dataset across the country (Secretaria de Salud 2021). As the COVID-19 pandemic started in 2020, which drove much higher death counts than in previous years, in our primary analyses we used data from 1998 to 2019, excluding 2020 data. In a set of sensitivity analyses, we included data for all available years. For municipality-level analysis, all municipalities with less than 500 deaths during the entire study period (1998–2019) were excluded (n = 718) to ensure results were not driven by outliers caused by small sample sizes. The excluded municipalities had an average of 2,788 (standard deviation (SD): 2,070) residents while included municipalities had an average of 63,414 (SD: 15,4297) residents.

Daily maximum and minimum 2-meter air temperature raster data were extracted from the Daymet V4: Daily Surface Weather and Climatological Summaries at a 1 km resolution for 1998 to 2020 (Thornton et al. 2022). This data source was selected because of its spatio-temporal resolution and availability in Mexico. The product uses interpolation and extrapolation of ground-based observations through statistical modeling techniques to provide gridded estimates of daily weather. Gridded 100×100m population grids obtained from WorldPop were reduced to the 1 km scale and raster multiplication was applied using the rgee package in R (Aybar et al. 2020; Tatem 2017). A reducer was used to estimate the population-temperature product and this was divided by the population of the municipality to estimate a population-weighted temperature values for each municipality and day. Each death was assigned the temperature of the municipality in which they died, which was used for the analyses at all scales (county, state, municipality).

Previous work has shown that local climatology and acclimation are important in health effects of heat, therefore we defined our main extreme heat exposures as a relative metric based on the local municipality temperature distribution. A day was considered an extreme heat day if it exceeded the 99th percentile of maximum temperature at the municipality-level during the study period (1998–2019 for main analysis). As the health effects of extreme heat are known to change when using different metrics to define the heatwave or extreme heat event (Xu et al. 2017), sensitivity analyses were conducted with seven extreme heat thresholds, considering both relative temperature thresholds (i.e., the hottest days within each municipality) and absolute thresholds (i.e., using a discrete temperature cutoff). For the relative thresholds we used 95th or 99th percentile for both the population-weighted minimum and maximum temperature distribution for the 1998–2019 period to define single day extreme heat events. Considering both the minima and maxima allowed us to include both daytime and nighttime extreme heat events. For the absolute indicators of extreme heat, we considered heat events to be days when the maximum temperature of the municipality was above 30 °C or 35 °C. At the country and state-level, we conducted additional sensitivity analyses evaluating 1-day and 2-day lag associations and 2-day extreme heat events (99th percentile).

Data on socio-demographics at the municipality level were collected from the 2010 Census of Population and Housing from the National Institute of Statistics and Geography of Mexico (INEGI 2010). Socio-demographic variables at the municipality level considered to be relevant for heat-related impacts were population size, percentage of persons without any schooling, percentage of persons who were illiterate, the median level of schooling, crowding (percentage with 3 or more people living per room in the household), percentage without electricity, percentage with no refrigerator or television, percentage with no amenities (no radio, television, refrigerator, washing machine, car, computer, fixed telephone, cell phone, or internet), percentage of women, percentage of population 65 + years of age, median age, and a marginalization score. The marginalization score was developed by the Mexican National Institute of Statistics and Geography (INEGI, for its acronym in Spanish) using information about a municipality’s educational level, health, housing, assets, and income to estimate the level of marginalization relative to other municipalities (INEGI 2010). Population density was estimated by calculating the number of persons per square kilometer for each municipality using land area estimated with the shapefile.

### 2.2. Statistical analysis

#### 2.2.1. Country and state-level analysis: time-stratified case-crossover design

We used a time-stratified case crossover design to study the association between extreme heat and mortality at the state-level across the 32 Mexican federal entities (31 states and Mexico City, the capital) (Lu and Zeger 2007). In this methodology, each mortality case serves as its own control. Control days were selected based on the same day of the week of the death within the same month and year that the case occurred. We used a conditional logistic regression model to study the association between extreme heat events and mortality in Mexico by state. A total of 33 separate models were run, one overall and for each state in Mexico, to evaluate the association between heat and mortality at the state level.

#### 2.2.2. Municipality-level analysis: within-community matched design

As some of the municipalities are small and have few days with deaths during the study period and our exposure is also rare, the time-stratified case crossover would likely not have sufficient heterogenous matched strata to detect an association at the municipality level if one existed. Therefore, to evaluate the effects of extreme heat at the municipality level, we applied a within-community matched design (Aguilera et al. 2020; Chen et al. 2024; Schwarz et al. 2021). The benefit of this approach is by including additional non-exposed days, we can aggregate additional information to study this association and improve precision of the result. The bayesian model further helps improve precision by including information from neighboring municipalities.

The within-community matched design approach considers each extreme heat day in each municipality as a specific event and identifies control days (within 60 days to control for seasonality and long-term trends) that are used as a comparison to estimate a measure of relative rate (RR) during exposed days as compared to days without extreme heat. All days that were more than 3 days away from each extreme heat day and within 60 days of each extreme heat day were used as controls, excluding any other extreme heat days that might have occurred within that window. A sensitivity analysis was conducted using 30 days as the control window. The 3-day window of exclusion before and after the extreme heat day serves to eliminate the potential lagged effect of heat from the selection of controls, the effects of extreme heat on mortality in Mexico have been shown to last 2–3 days and then subside (Cohen and Dechezleprêtre 2022). A sensitivity analysis was conducted considering a 6-day time window for exclusion. An inverse time weighting scheme (i. e., weight equal to 1/distance to exposed day) was applied in which control days closer in time to the exposed day were given a stronger weight than those that were further. An RR for each extreme heat event was then calculated by taking the total number of deaths on the day of the extreme heat event divided by the inverse-time weighted average of deaths on all control days. A municipality-level measure of RR was estimated by taking an average for all the extreme heat events in each municipality. We then estimated excess relative risk (ERR) by subtracting 1 from the RR.

#### 2.2.3. Spatial Bayesian hierarchical model

We used spatial information to increase the precision of our municipality-level results and account for spatial autocorrelation. Similar to previously published work by coauthors, we used a spatial BHM for this purpose (Aguilera et al. 2020; Schwarz et al. 2021). The ERR estimates for all municipalities obtained from the within-community matched design analysis were used as the response variable in a spatial linear model. The BHM was fit using the *spBayes* package in R (Finley et al. 2007). We computed population-weighted centroids for each municipality in Mexico using Worldpop 1 km grids from 2010 (Tatem 2017), which were then used as input for the model fit with *spBayes*. A spherical covariance function was used. We fit an empirical semivariogram to estimate the starting values for the spatial parameters: sill (σ2), nugget (τ2), and range (ϕ). Tuning parameters were approximated based on the semivariogram. Flat priors were used for the modelling to allow for maximum flexibility. Monte Carlo Markov sampling was applied with 10,000 samples, and 75 % were used for burn-in, similar to previous research applying this approach (Chen et al. 2024; Schwarz et al. 2021). To estimate the statistical precision of the point estimates, we computed the signal-to-noise ratio (SNR) from the model output, which represents the ratio between the mean and standard deviation of recovered samples. The SNR was mapped for each municipality to represent statistical precision. This gives a visual representation of areas where estimates of ERR are precise. A conventional cutoff was used of |SNR| > 2 to represent acceptable precision. This methodology assumes isotropy, or a homogenous spatial autocorrelation in all directions. The BHM is used to capitalize on spatial autocorrelation in effect estimates and increase precision, and was applied to improve municipality specific estimates from the within-community matched design.

#### 2.2.4. Meta-regression

A *meta*-regression was applied using these spatially improved estimates of ERR from the BHM as the outcome to understand what municipality-level factors were important predictors in the spatial heterogeneity in impacts of heat. Each socio-demographic variable was separately included in a meta*-*regression model, using the spatial estimate and variance from the BHM to estimate changes in association for each inter-quartile range (IQR) in the socio-demographics to increase comparability. Effect estimates and standard deviation were taken from each model to represent the importance of a change in IQR of each variable in predicting spatial distribution of heat effects across Mexico. For reproducibility purposes, sample datasets and code used for this project are provided in the following repository: https://github.com/benmarhnia-lab/spatial_heat_Mexico.

## 3. Results

**Fig. 1.**
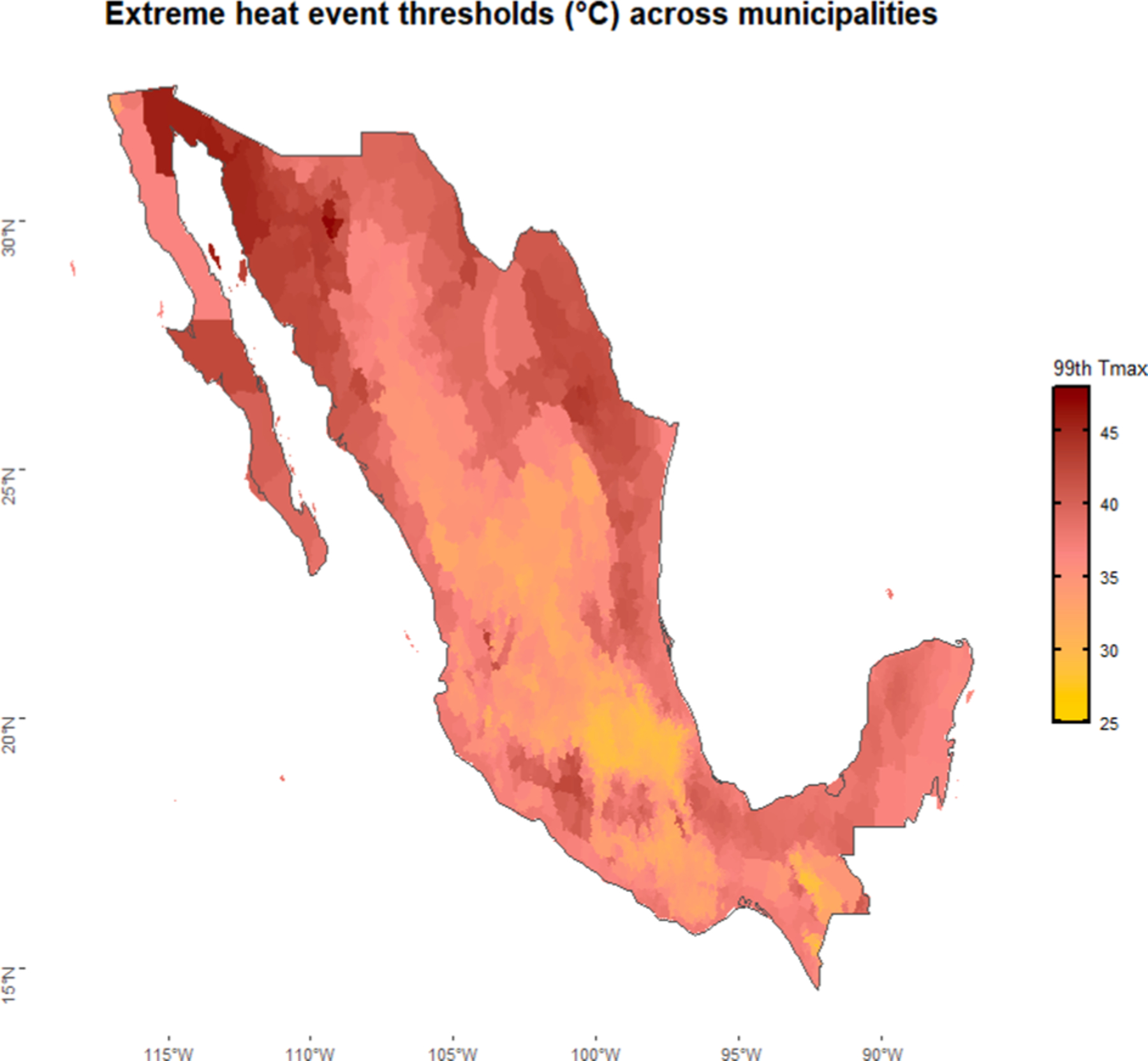
Municipality-level thresholds used to define extreme heat events (99th percentile of daily temperature) across Mexico, 1998–2019.

The average daily mortality count across all municipalities was 0.62 on non-heat days and was higher on days of extreme heat and higher on extreme heat days (0.66) based on the 99th percentile definition (Table 1). Information about the age and gender distribution of deaths is included in Sup. Table S1. The temperature distribution varies by state and municipality, with the warmer months usually falling between March and October (Supplementary Fig. S1). At the 99th percentile, the temperature threshold for daytime extreme heat events ranges between 25 °C and 45 °C (Fig. 1).

**Fig. 2.**
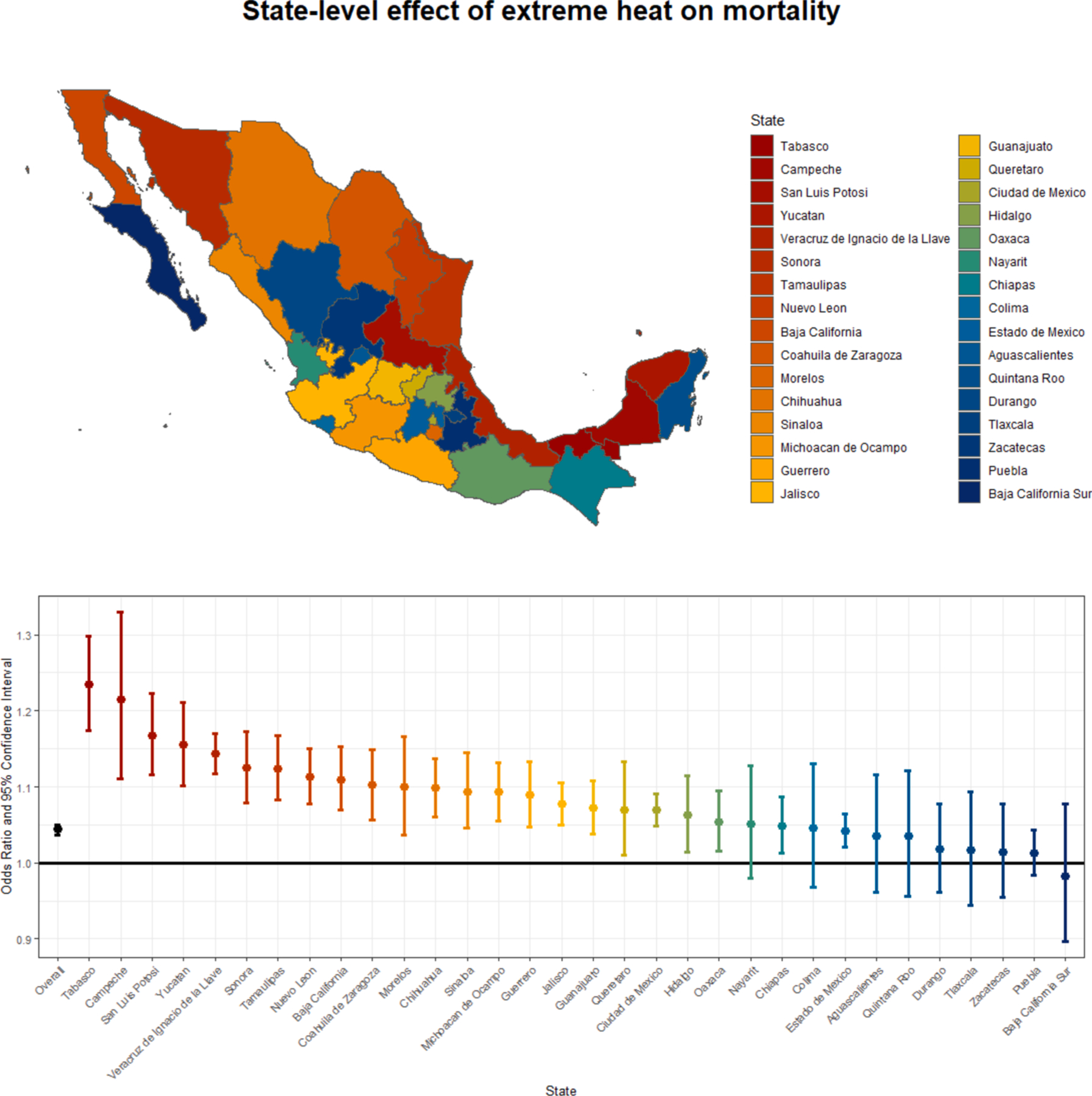
Overall and state-level odds ratios and 95% confidence intervals (CI) of the effect of extreme heat (99th percentile maximum temperature of municipality of death) on mortality in Mexico using a time-stratified case-crossover design, 1998–2019.

With the time-stratified case crossover analysis, we observed an effect of extreme heat on all-cause mortality in Mexico. Extreme heat days increased mortality by 1.083 [95 % CI: 1.076, 1.09] across the country. This was consistent across sensitivity analyses considering differing thresholds for extreme heat (Sup. Table S2). There is variation in the effects of heat across states, with Tabasco showing the greatest effects of heat (Fig. 2). The state-level analysis showed consistent results across different thresholds used in the sensitivity analysis, including lagged and 2-day associations, although there was some variation in precision and strength of estimates across states. The standard deviation state-level coefficient was wider than what would be expected from sampling variation (standard deviation of state level coefficients = 0.15, standard error in overall model = 0.0034). Generally, the eastern coastal regions of Mexico show greater effects of extreme heat.

**Fig. 3.**
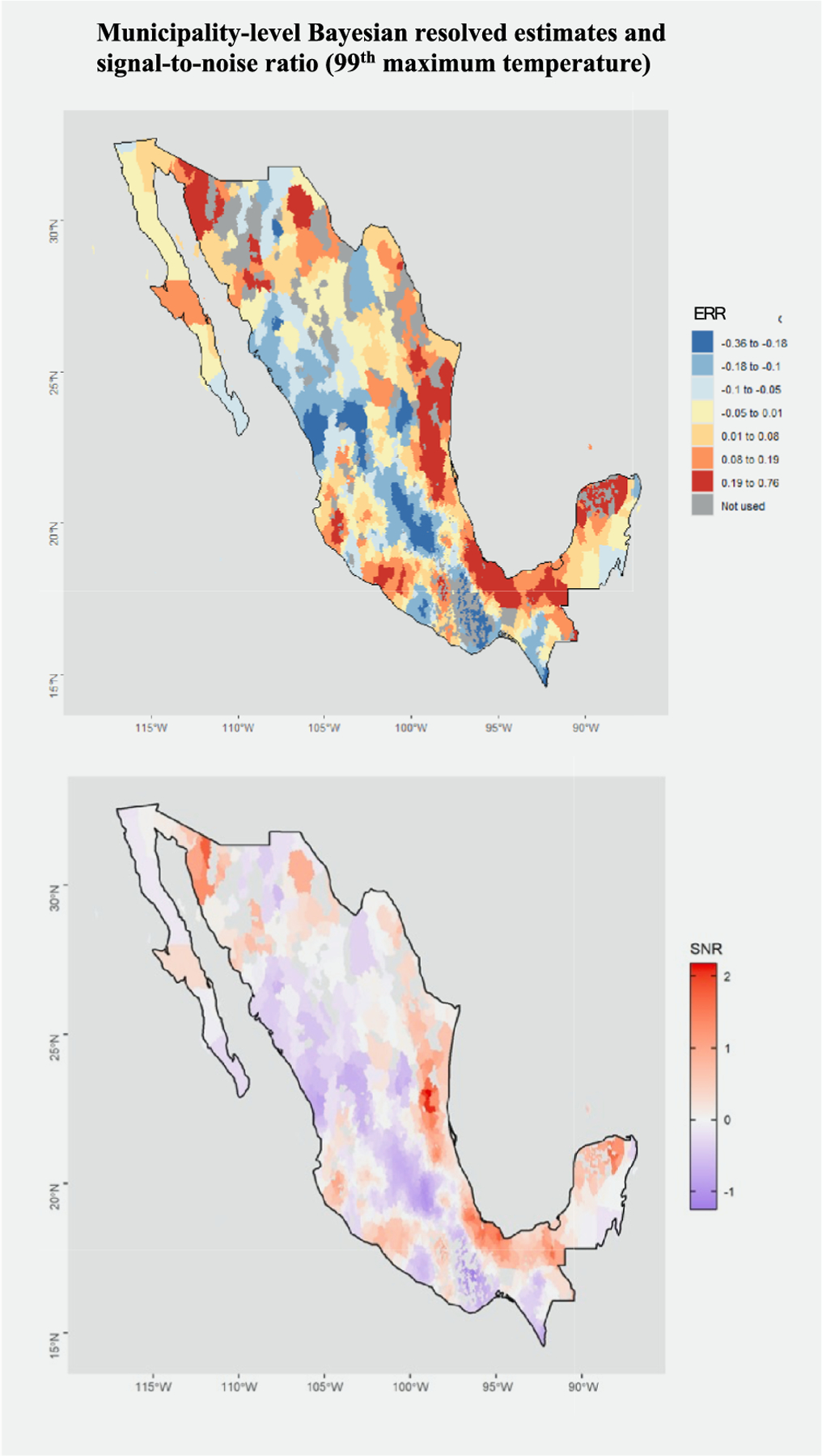
Bayesian resolved estimates of the excess relative risk (top) and signal-to-noise (SNR) ratio (bottom) of the effect of daytime extreme heat events (99th percentile maximum temperature) on all-cause mortality across Mexico, 1998–2019.

Results of the within-community matched design at the municipality-level showed substantial spatial heterogeneity in the effects of extreme heat, including some areas showing an ERR of up to 0.76 with other areas even showing a negative ERR (Fig. 3). When considering the municipality-level estimates, precision varies geographically and by extreme heat measure, with few municipalities showing significant values (defined by |SNR| > 2) (Supplementary Fig. S3). We observe particularly strong effects of extreme heat in the eastern coastal regions of Mexico as well as some hotspots on the western coast. Results were consistent across all measures of extreme heat and in the sensitivity analysis including 2020 (Supplementary Figs. S3, 4 & 5). Results were also consistent when changing the control selection to a 30-day window and changing exclusion days to 6 (Supplementary Fig. S6).

**Fig. 4.**
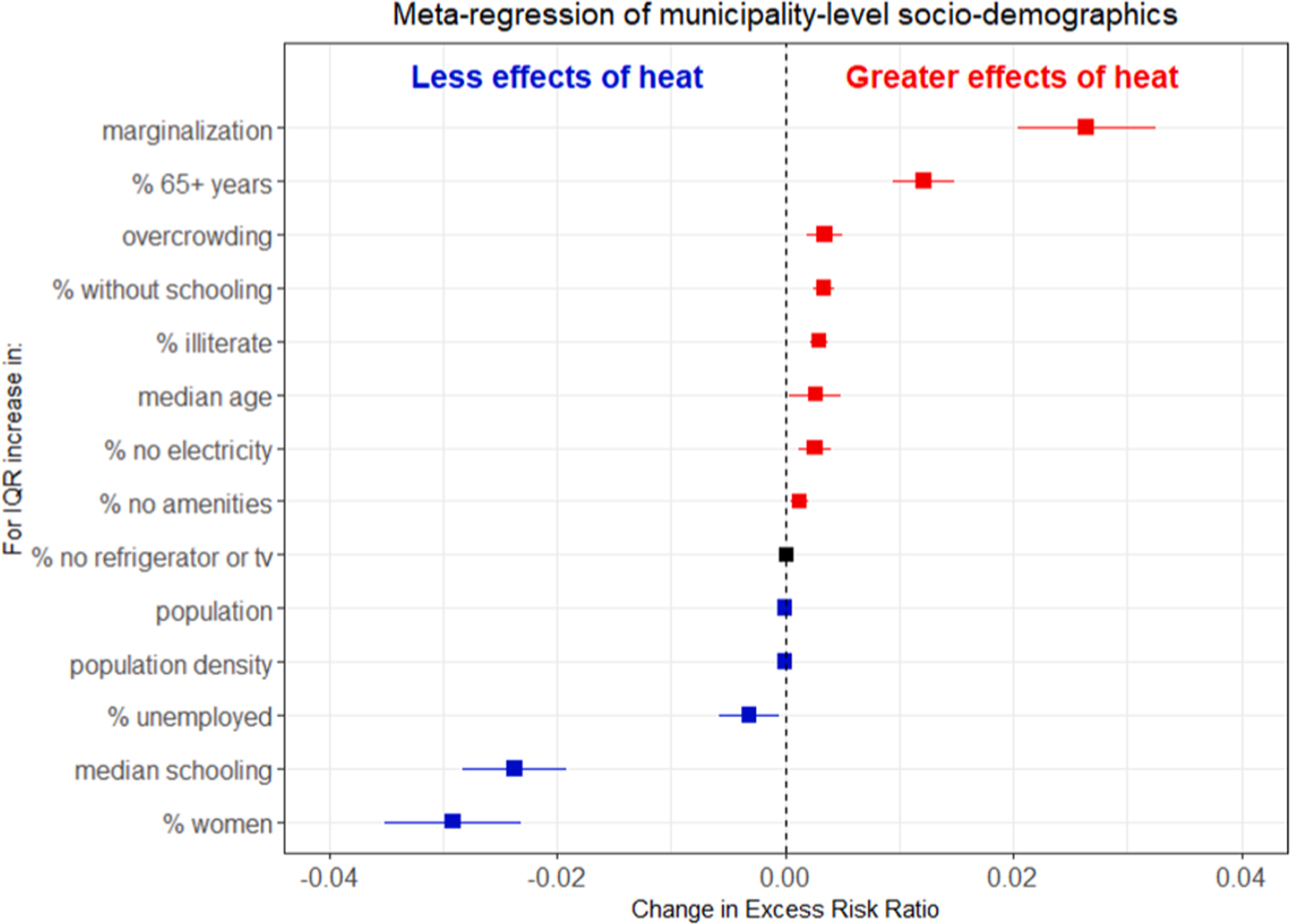
Results of meta-regression of the predictors of spatial variation in the effect of daytime extreme heat events (99th percentile maximum temperature) on all-cause mortality across Mexico, 1998–2019.

Socio-demographic characteristics vary across Mexico with coastal regions showing higher marginalization (Supplementary Fig. S7). Many of the socio-demographics are highly correlated, yet we consider them each individually to understand their specific role in predicting heat-related effects across municipalities (Supplementary Fig. S8). We find an increased risk of heat-related effects in socially vulnerable municipalities. Education level is inversely predictive of heat-related effects—as a municipality’s education level increases, decreased effects of extreme heat are observed (Fig. 4). The effect of extreme heat on mortality is greater in municipalities that have a higher percentage of individuals without schooling and a higher percentage of illiteracy, and the impacts are less in municipalities with a higher median schooling level. This is consistent across relative measures of extreme heat (Supplementary Fig. S9).

Similar trends are found for certain housing-related characteristics; a higher percentage of crowded households (3 + persons per room) and households with no electricity increased heat-related risks for extreme heat metrics on the relative scale (Fig. 4). The percentage of households without a television or refrigerator, a measure of household wealth, and the percentage of households with no amenities were not important predictors of heat-related impacts. Also, results suggest municipalities with higher unemployment show decreased heat-related impacts for relative measures of extreme heat. Municipalities with a higher percentage of women also showed decreased effects of extreme heat (Fig. 4). Municipalities with a higher median age and a greater percentage of residents over 65 years of age are at increased risk of heat-related impacts. Lastly, a higher marginalization is the strongest predictor explaining the greater effects of heat (at the 99th percentile of maximum temperature) on mortality.

## 4. Discussion

This study used a multi-scale analysis to quantify the effects of extreme heat events on mortality at the country level, state-level and municipality-level. In the municipality-level analysis, we examined heterogeneity in heat-related health impacts across municipalities and explored which vulnerability factors are important predictors in the observed variation. The results are three-fold. First, we find that extreme heat increases the risk of mortality in Mexico. Second, state-level analysis shows considerable spatial heterogeneity in the effects of extreme heat on mortality, with most states of Mexico showing increased risk. Lastly, there are substantial differences in the effects of heat at the municipality level and socio-demographics such as the level of marginalization, median age and education of municipalities appear to be important predictors of the differences we observed across Mexico, indicating that social vulnerability factors were associated with increased risk of heat-related effects.

We found that extreme heat drives an increased odds of mortality for extreme heat events in Mexico overall. The positive association between extreme heat and mortality we found is consistent with many previous studies demonstrating the pervasive effects of extreme heat on a global scale (Cheng et al. 2019; Ebi et al. 2021; Green et al. 2019) and previous studies showing the effects of extreme heat in Mexico (Cohen and Dechezleprêtre 2022; Guerrero Compeán, 2013; Tobías et al. 2024). Cohen and Dechezleprêtre (2022) found 2 percent of deaths from 1998 to 2017 to be induced by hot temperatures on days over 32 °C (Cohen and Dechezleprêtre 2022). Our results indicate a 1.061 [95 % CI: 1.057, 1.065] higher odds of mortality from extreme heat for temperatures above 35 °C. We observe substantial variation in heat-related impacts across Mexico for measures of extreme heat on the relative and absolute scales in our sensitivity analyses while all associations remained positive, confirming that exposure to extreme heat increases mortality (Supplementary Fig. 2; Sup. Table S3).

The observed effects of extreme heat on mortality are not uniform across Mexico and show substantial spatial heterogeneity for both the state and municipality resolutions (Figs. 2 & 3). Northern and eastern states in Mexico are particularly affected by heat, with odds ratios up to 1.23 (95 % CI: 1.17, 1.30) in the southeastern state of Tabasco (Fig. 2). We notably found that eastern, southwestern and Sonora coastal regions of Mexico had higher heat-related effects (Figs. 2 & 3); this is consistent with recent work evaluating city-level effects of extreme temperature on mortality in Latin America which found particularly high effects in coastal cities (Kephart et al. 2022). The vulnerability of coastal regions to the effects of heat has also been observed in other areas including San Diego County, where lack of adaptation and acclimatization, as well as lower air conditioning rates, are likely drivers of this vulnerability as populations have a lower coping capacity to deal with these hazards since extreme heat is rarer along the coast than inland regions (Guirguis et al. 2018). Similar drivers may explain the heterogeneity in Mexico, and future research could consider the role of adaptation and acclimatization in this association. Another reason may be the differing regional topography of Mexico with more mountainous regions inland that may be less affected by heat.

At the municipality scale, we observed a wide range of impacts across municipalities with some regions showing a negative ERR (although the effects are not precise) and other regions showing an ERR up to 0.76 (for 99th percentile maximum temperature extreme heat events). Understanding municipality-level effects can be important to effectively estimate the expected burden of extreme heat, increase preparedness and prioritize vulnerable communities. For example, municipalities with observed high effect of heat on mortality could be prioritized the design and implementation of heat action plans. This can include actions such as the dissemination of heat advice on how to best protect oneself during an extreme heat event, sending alerts, and ensuring cooling centers are accessible, particularly for those that don’t have access to air conditioning which can be critical in reducing the burden of heat (Bedi et al. 2022; Lowe et al. 2011). Informing heat action plans that are activated based on epidemiological evidence is most effective in reducing the burden of heat-related health impacts (Benmarhnia et al. 2019).

We also identified that spatial heterogeneity in heat-related effects was predicted by socio-demographics. Municipality-level socio-economic population characteristics such as education and income can be important in explaining spatial variations in health risk that are observed across regions (Chen et al. 2024; Schwarz et al. 2021). The strongest social factor in predicting municipality-level differences in heat effects across Mexico was the marginalization index, indicating that municipalities with higher marginalization are at a higher risk of mortality from extreme heat. This is consistent with previous work showing that lower community-level SES may increase heat-related mortality risk (Son et al. 2019).

Similarly to previous studies, the higher the median age of a municipality and the higher percentage of persons above age 65, the higher risk it had for heat-related effects. This is expected, as older populations are known to be particularly vulnerable to the adverse effects of heat. A systematic review investigating vulnerability to heat-related mortality revealed a ratio of relative risk, or increased risk, of 1.02 (95 % CI: 1.01, 1.03) for persons aged > 65 years when compared to younger age groups, similar to our findings, and 1.04 (95 % CI = 1.02, 1.07) for ages > 75 years (Benmarhnia et al., 2015). The vulnerability of populations with higher proportions of older adults may be due to their physiologic challenges to thermoregulate and their higher prevalence of underlying medical conditions that can limit their ability to physiologically respond to heat (Son et al. 2019). Mental disorders can also alter risk perceptions and older individuals may be less likely to take precautions against the effects of heat (Åström et al. 2011).

Municipalities with more overcrowded households showed increased heat effects on the relative scale, while the opposite was observed for extreme heat metrics on the absolute scale. Overcrowding and house-hold wealth were not included as indicators in a systematic review of effect modifiers of temperature-related mortality, but weak evidence was found related to poor housing quality (Son et al. 2019). Our results suggest that municipalities with overcrowded households may be important to target and prioritize in heat action plans. The population size of a municipality was not found to be associated with heat effects either. This is consistent with previous work that showed similar vulnerability to heat for rural and urban municipalities (Cohen and Dechezleprêtre 2022).

Municipality-level education was also found to be predictive of heat-related impacts for relative measures of extreme heat with increased effects in municipalities with a higher percentage of the population with no schooling or higher illiteracy and decreased effects in municipalities with higher median schooling. Lower education may be associated with lower risk perception and protective behaviors and is also strongly linked with SES (Son et al. 2019). Previous work from New York City has shown that individuals with a lower income may be less likely to be aware of heat-related risks, although they had a higher concern for the impacts of climate change on their health (Madrigano et al. 2018). Also, populations from a lower SES may be more likely to work outdoors and have more direct exposure to extreme heat. Notably, previous work did not identify individual-level education as an effect modifier between heat and mortality in Mexico City (Bell et al. 2008). A systematic review found limited evidence of higher risks of heat from lower education although 16 out of the 26 studies included in the review found a higher risk for those with no or lower education (Son et al. 2019). Our results add to this evidence by demonstrating that municipality-level educational indicators can be important in predicting an increased risk of heat in Mexican municipalities, particularly when considering relative measures of heat. More research would have to be conducted to understand these mechanisms and how to prioritize these communities in measures to reduce the burden of heat in communities that are already at-risk.

Interestingly, municipalities with a higher unemployment rate showed lower heat-related effects using relative extreme heat measures, but increased effects when using absolute temperature over 30°C (Supplementary Fig. S9). Previous work shows that unemployment can increase the risk of heat-related impacts (Anderson and Bell 2009; Leone et al. 2013). Further work should evaluate how social vulnerability to relative and absolute measures varies to understand these nuanced differences in effects and inform effective adaptation measures. Understanding the role of acclimation and acclimatization at the individual-and community-level and mechanisms that drive differences in heat vulnerability will be essential to protect the most vulnerable in the context of climate change.

The percentage of households without television or refrigerator, a measure of household wealth, was not an important predictor of heat-related impacts for any measure, nor was the percentage of house-holds with no amenities. Additionally, municipalities with a higher percentage of women showed lower impacts of extreme heat on the relative scale while municipalities with more women were more affected by extreme heat on the absolute scale. Previous research shows that women may be more vulnerable to heat impacts (Son et al. 2019). Our results may indicate that relative and absolute measures of extreme heat are different concepts that may have varying impacts and should be further studied. Studying the role of racism/colorism in heat-related vulnerability in the context of Mexico would also be an interesting and important question to explore in future work.

The combination of these results indicate that uniform exposure metrics and effect risks may not be sufficient to capture the full extent of heat-related impacts for a country and highlights the importance of considering spatial variation in the exposure measure and effects at different scales. This is consistent with previous work using a similar methodology to understand the spatial variation in joint effects of heat and ozone pollution on respiratory hospitalizations across California zip codes (Schwarz et al. 2021). This adds to the growing literature showing the importance of investigating fine-scale spatial differences in the health effects of heat (Hondula et al. 2012; Song et al. 2021; Wang et al. 2021). Studying these spatial differences and using them to inform policies has been shown to be effective in improving the effectiveness of heat action plans and public health interventions to reduce the burden of extreme heat (Benmarhnia et al. 2019; Carmona et al. 2017).

To our knowledge, this is the first study to explore multi-level spatial variation in heat-related effects in Mexico and highlight socio-demographics at the municipality level that are predictors in the observed variation. There are several strengths to this study. By applying a multi-stage analysis using a within-community matched design with spatial BHM and meta-regression, we capitalize on population-weighted temperature measures at the fine spatial scale to understand the differential role of extreme heat across populations. Additionally, by using publicly available satellite imagery and datasets that are available on an online repository, we develop a protocol that can be replicated in other contexts and regions to further understand how heat impacts vary spatially within and across different settings.

Nevertheless, there are also limitations to the data sources and approach that should be acknowledged. First, we focus this study on extreme heat events based on maximum and minimum temperature, but other weather-related drivers such as humidity may also be important in explaining differences in impacts. Also, the mortality data is from the national statistics for Mexico, and previous research using this data has indicated that official records in Mexico were thought to underestimate deaths by 13.7 % (Bell et al. 2008; Silvi 2003). However, it was later shown that Mexico had 90 % or more complete death registration and their certification system follows international standards (Braine 2006). The Bayesian model assumes isotropy, considering the distance of spatial autocorrelation to be uniform in all directions, which may not hold as elevation changes and other geographical factors may lead to violation of this assumption in areas with sudden changes to topography. Also, by focusing the analysis on municipalities with more than 500 deaths for the municipality-level analysis, we may be excluding rural areas where the impacts of extreme heat effects may be worse. However, the population size of a municipality did not appear to be an important predictor of heat impacts in this context. Also, we consider only same-day events in our municipality-level analysis, while there may be mortality displacement. However, we continue to observe a positive association up to 2 days following the extreme heat days in our country and state-level analyses, so this may not be an important factor in this context. Lastly, there may still be residual confounding in the observed associations. Considering the role of humidity, air pollution and wildfire smoke as effect modifiers would also be an interesting area for future research.

The adverse effects of extreme heat on public health are well established. However, for many regions around the globe, it is unclear whether the impacts may be worse for specific populations or marginalized sub-populations. Countries that contribute the least to anthropogenic climate change will be the most affected by its impacts (Bathiany et al. 2018; Gasparrini et al. 2017); yet there is substantially less evidence coming from many of these regions (Green et al. 2019). This could impair efforts to mitigate the effects of extreme heat on population health. Understanding the specific impacts of extreme heat at various scales is important due to the heterogeneity in the impacts of extreme heat. We found unique municipality-level vulnerability factors in Mexico, such as education and gender, that differ from existing evidence in other settings. In addition, processes of social marginalization vary substantially around the globe due to historical and geopolitical factors, and the plausible state- or national-level climate health interventions need to be tailored to fit those contexts. Characterizing differential vulnerability to extreme heat is critical to estimating the epidemiological impacts of these events in understudied regions of the world and, ultimately, to designing and implementing interventions to mitigate the growing harms from climate-driven extreme heat events.

## Supplementary Material

Supplementary appendix

## Figures and Tables

**Table 1:** Descriptive statistics of mortality, heat events, and socio-demographics across Mexican municipalities, 1998–2019.

Mortality and Climate Conditions					
Extreme heat event	Threshold (°C) Mean (IQR)	Extreme heat days Total count	Daily mortality count (mean)		
			Extreme heat days	Non-heat days	t-test
99th maximum	35.93	199,017	0.66	0.62	p <
	(5.63)				0.0001
95th maximum	34.35	987,714	0.64	0.62	p <
	(5.59)				0.0001
99th minimum	19.43	199,017	0.70	0.62	p <
	(8.87)				0.0001
95th minimum	18.55	987,714	0.67	0.62	p <
	(8.74)				0.0001
>30 °C	30	7,064,298	0.65	0.56	p <
					0.0001
>35 °C	35	1,446,652	0.67	0.62	p <
					0.0001
Socio-demographics					
	Median	IQR	Min	Max	
Population	21,275	34,714	2458	1,815,786	
Women (%)	51.0	1.7	46.4	55.7	
Unemployment (%)	3.9	2.9	0.2	37.4	

Without schooling (%)	10.7	9.9	0.9	56.0	

Illiteracy (%)	11.1	11.0	0.6	58.7	
Crowding* (%)	4.7	5.0	0.3	37.3	
No electricity (%)	2.4	3.5	0	64.9	

No refrigerator or tv (%)	19.0	26.5	0.6	64.9	

No amenities (%)	3.5	6.8	0.1	57.6	
Age 65 + years (%)	7.2	3.3	1.3	20.4	

Median age	25.0	4.0	22.0	36.0	
Marginalization^#^	3.0	2.0	1.0	5.0	

*Crowding = 3 + people per room.

^#^ 1-very low, 2-low, 3- medium, 4-high, 5-very high.

## Data Availability

Sample code and materials used for this manuscript are available here: https://github.com/benmarhnia-lab/spatial_heat_Mexico
