# Supplementary material for: Heat-related mortality in Mexico: A multi-scale spatial analysis of extreme heat effects and municipality-level vulnerability
Source: Environ Int. Author manuscript; Available in PMC 2025 Apr 30. (PMC12043324; doi:10.1016/j.envint.2024.109231)
Supplement: Supplementary appendix [file NIHMS2072337-supplement-Supplementary_appendix.docx]

**Title:** Heat-related mortality in Mexico: a multi-scale spatial analysis of extreme heat effects and municipality-level vulnerability

**Supplementary Appendix**

**Supplementary Table 1:** Age and gender distribution of deaths in Mexico, 1998-2020.

|  | **Number of deaths** | **% deaths** |
| --- | --- | --- |
| **Age** |  |  |
| <1 | 695,213 | 5.18 |
| 1-19 | 47,9640 | 3.57 |
| 20-44 | 1,770,699 | 13.19 |
| 45-64 | 3,135,097 | 23.35 |
| 65+ | 7,279,009 | 54.21 |
| unspecified | 67,273 | 0.50 |
| **Sex** |  |  |
| women | 5,891,093 | 43.88 |
| men | 7,528,371 | 56.07 |
| unspecified | 7,467 | 0.05 |
| Total | 13,426,931 | 100 |

**Supplementary Table 2:** Results of case-crossover analysis of overall effect of extreme heat on mortality in Mexico for main analysis (99^th^ maximum) and sensitivity analysis using different measures of extreme heat events on the relative and absolute scales.

| **Extreme heat** | **OR** | **95% CI** |
| --- | --- | --- |
| 99^th^ maximum | 1.083 | [1.076, 1.09] |
| 95^th^ maximum | 1.061 | [1.057, 1.064] |
| 99^th^ minimum | 1.044 | [1.037, 1.05] |
| 95^th^ minimum | 1.022 | [1.018, 1.025] |
| > 30°C | 1.028 | [1.025, 1.03] |
| > 35°C | 1.061 | [1.057, 1.065] |
| 2-day 99^th^ maximum | 1.088 | [1.078, 1.099] |
| 1-day lag 99^th^ maximum | 1.071 | [1.063, 1.078] |
| 2-day lag 99^th^ maximum | 1.05 | [1.043, 1.057] |

**Supplementary Table 3:** Odds ratios and confidence intervals for sensitivity analysis at state-level of differing extreme heat metrics, 1998-2019.

| **state** | **state name** | **extreme heat event** | **OR** | **lower_ci** | **upper_ci** |
| --- | --- | --- | --- | --- | --- |
| 1 | Aguascalientes | hw_99 | 1.036 | 0.962 | 1.115 |
| 1 | Aguascalientes | hw_95 | 1.046 | 1.008 | 1.085 |
| 1 | Aguascalientes | hw_99_min | 0.985 | 0.920 | 1.056 |
| 1 | Aguascalientes | hw_95_min | 0.987 | 0.952 | 1.024 |
| 1 | Aguascalientes | hw_30abs | 1.050 | 1.022 | 1.078 |
| 1 | Aguascalientes | hw_35abs | 1.092 | 0.935 | 1.275 |
| 1 | Aguascalientes | hw_99_lag1 | 1.028 | 0.954 | 1.108 |
| 1 | Aguascalientes | hw_99_lag2 | 1.030 | 0.955 | 1.110 |
| 1 | Aguascalientes | hw_99_2 | 0.973 | 0.883 | 1.072 |
| 1 | Aguascalientes | hw_99 | 1.036 | 0.962 | 1.115 |
| 2 | Baja California | hw_99_min | 1.138 | 1.094 | 1.184 |
| 2 | Baja California | hw_95_min | 1.061 | 1.040 | 1.083 |
| 2 | Baja California | hw_30abs | 1.051 | 1.034 | 1.067 |
| 2 | Baja California | hw_99 | 1.110 | 1.069 | 1.152 |
| 2 | Baja California | hw_95 | 1.085 | 1.065 | 1.106 |
| 2 | Baja California | hw_99 | 1.110 | 1.069 | 1.152 |
| 2 | Baja California | hw_99_2 | 1.154 | 1.090 | 1.220 |
| 2 | Baja California | hw_35abs | 1.041 | 1.016 | 1.067 |
| 2 | Baja California | hw_99_lag1 | 1.121 | 1.080 | 1.164 |
| 2 | Baja California | hw_99_lag2 | 1.121 | 1.080 | 1.164 |
| 3 | Baja California Sur | hw_99 | 0.983 | 0.897 | 1.077 |
| 3 | Baja California Sur | hw_95 | 1.047 | 0.996 | 1.101 |
| 3 | Baja California Sur | hw_99_min | 0.936 | 0.850 | 1.030 |
| 3 | Baja California Sur | hw_99_2 | 0.936 | 0.806 | 1.088 |
| 3 | Baja California Sur | hw_30abs | 0.993 | 0.960 | 1.027 |
| 3 | Baja California Sur | hw_35abs | 1.059 | 1.024 | 1.096 |
| 3 | Baja California Sur | hw_99_lag1 | 1.047 | 0.957 | 1.145 |
| 3 | Baja California Sur | hw_95_min | 0.962 | 0.910 | 1.016 |
| 3 | Baja California Sur | hw_99 | 0.983 | 0.897 | 1.077 |
| 3 | Baja California Sur | hw_99_lag2 | 1.017 | 0.928 | 1.116 |
| 4 | Campeche | hw_99_min | 1.195 | 1.104 | 1.293 |
| 4 | Campeche | hw_99 | 1.215 | 1.110 | 1.329 |
| 4 | Campeche | hw_95 | 1.112 | 1.064 | 1.162 |
| 4 | Campeche | hw_95_min | 1.028 | 0.987 | 1.070 |
| 4 | Campeche | hw_30abs | 1.040 | 1.011 | 1.069 |
| 4 | Campeche | hw_35abs | 1.069 | 1.043 | 1.097 |
| 4 | Campeche | hw_99_lag1 | 1.158 | 1.057 | 1.268 |
| 4 | Campeche | hw_99_lag2 | 1.087 | 0.992 | 1.191 |
| 4 | Campeche | hw_99_2 | 1.249 | 1.104 | 1.413 |
| 4 | Campeche | hw_99 | 1.215 | 1.110 | 1.329 |
| 5 | Coahuila de Zaragoza | hw_99 | 1.102 | 1.057 | 1.149 |
| 5 | Coahuila de Zaragoza | hw_95 | 1.080 | 1.056 | 1.104 |
| 5 | Coahuila de Zaragoza | hw_99_min | 1.070 | 1.025 | 1.116 |
| 5 | Coahuila de Zaragoza | hw_30abs | 1.024 | 1.009 | 1.039 |
| 5 | Coahuila de Zaragoza | hw_35abs | 1.065 | 1.047 | 1.083 |
| 5 | Coahuila de Zaragoza | hw_99_lag1 | 1.086 | 1.042 | 1.133 |
| 5 | Coahuila de Zaragoza | hw_95_min | 1.056 | 1.033 | 1.080 |
| 5 | Coahuila de Zaragoza | hw_99 | 1.102 | 1.057 | 1.149 |
| 5 | Coahuila de Zaragoza | hw_99_2 | 1.076 | 1.011 | 1.145 |
| 5 | Coahuila de Zaragoza | hw_99_lag2 | 1.058 | 1.014 | 1.104 |
| 6 | Colima | hw_99 | 1.046 | 0.968 | 1.130 |
| 6 | Colima | hw_95 | 1.032 | 0.991 | 1.076 |
| 6 | Colima | hw_99_min | 1.070 | 0.995 | 1.151 |
| 6 | Colima | hw_30abs | 1.041 | 1.002 | 1.080 |
| 6 | Colima | hw_35abs | 1.072 | 1.039 | 1.105 |
| 6 | Colima | hw_99_lag1 | 0.990 | 0.916 | 1.070 |
| 6 | Colima | hw_95_min | 1.015 | 0.976 | 1.056 |
| 6 | Colima | hw_99_2 | 1.001 | 0.856 | 1.171 |
| 6 | Colima | hw_99_lag2 | 1.048 | 0.971 | 1.131 |
| 6 | Colima | hw_99 | 1.046 | 0.968 | 1.130 |
| 7 | Chiapas | hw_99 | 1.049 | 1.013 | 1.087 |
| 7 | Chiapas | hw_95 | 1.054 | 1.035 | 1.073 |
| 7 | Chiapas | hw_99 | 1.049 | 1.013 | 1.087 |
| 7 | Chiapas | hw_35abs | 1.065 | 1.046 | 1.084 |
| 7 | Chiapas | hw_99_lag1 | 1.023 | 0.988 | 1.060 |
| 7 | Chiapas | hw_99_lag2 | 1.000 | 0.965 | 1.036 |
| 7 | Chiapas | hw_99_2 | 1.036 | 0.983 | 1.092 |
| 7 | Chiapas | hw_30abs | 1.014 | 1.002 | 1.027 |
| 7 | Chiapas | hw_99_min | 1.006 | 0.972 | 1.041 |
| 7 | Chiapas | hw_95_min | 1.017 | 1.000 | 1.035 |
| 8 | Chihuahua | hw_95 | 1.073 | 1.053 | 1.093 |
| 8 | Chihuahua | hw_99 | 1.098 | 1.061 | 1.137 |
| 8 | Chihuahua | hw_35abs | 1.059 | 1.044 | 1.075 |
| 8 | Chihuahua | hw_99_min | 1.076 | 1.039 | 1.115 |
| 8 | Chihuahua | hw_95_min | 1.051 | 1.031 | 1.071 |
| 8 | Chihuahua | hw_30abs | 1.029 | 1.017 | 1.042 |
| 8 | Chihuahua | hw_99 | 1.098 | 1.061 | 1.137 |
| 8 | Chihuahua | hw_99_lag1 | 1.099 | 1.061 | 1.138 |
| 8 | Chihuahua | hw_99_lag2 | 1.115 | 1.077 | 1.154 |
| 8 | Chihuahua | hw_99_2 | 1.120 | 1.069 | 1.173 |
| 9 | Distrito Federal | hw_99_min | 1.009 | 0.989 | 1.029 |
| 9 | Distrito Federal | hw_99_lag1 | 1.032 | 1.012 | 1.053 |
| 9 | Distrito Federal | hw_95_min | 0.999 | 0.990 | 1.009 |
| 9 | Distrito Federal | hw_30abs | 1.045 | 1.033 | 1.058 |
| 9 | Distrito Federal | hw_35abs | NA | NA | NA |
| 9 | Distrito Federal | hw_99 | 1.070 | 1.049 | 1.091 |
| 9 | Distrito Federal | hw_99_lag2 | 1.017 | 0.997 | 1.038 |
| 9 | Distrito Federal | hw_99_2 | 1.058 | 1.030 | 1.086 |
| 9 | Distrito Federal | hw_99 | 1.070 | 1.049 | 1.091 |
| 9 | Distrito Federal | hw_99 | 1.070 | 1.049 | 1.091 |
| 9 | Distrito Federal | hw_95 | 1.038 | 1.027 | 1.049 |
| 9 | Distrito Federal | hw_99 | 1.070 | 1.049 | 1.091 |
| 10 | Durango | hw_99 | 1.018 | 0.961 | 1.077 |
| 10 | Durango | hw_99_min | 1.035 | 0.977 | 1.097 |
| 10 | Durango | hw_95_min | 1.012 | 0.982 | 1.043 |
| 10 | Durango | hw_30abs | 1.021 | 1.001 | 1.042 |
| 10 | Durango | hw_35abs | 1.042 | 1.010 | 1.075 |
| 10 | Durango | hw_99_lag1 | 1.095 | 1.035 | 1.158 |
| 10 | Durango | hw_99_lag2 | 1.011 | 0.955 | 1.071 |
| 10 | Durango | hw_99_2 | 1.080 | 1.004 | 1.163 |
| 10 | Durango | hw_95 | 1.046 | 1.015 | 1.078 |
| 10 | Durango | hw_99 | 1.018 | 0.961 | 1.077 |
| 11 | Guanajuato | hw_99 | 1.072 | 1.038 | 1.108 |
| 11 | Guanajuato | hw_95 | 1.065 | 1.048 | 1.083 |
| 11 | Guanajuato | hw_30abs | 1.042 | 1.031 | 1.054 |
| 11 | Guanajuato | hw_35abs | 1.083 | 1.035 | 1.133 |
| 11 | Guanajuato | hw_99_min | 1.023 | 0.991 | 1.055 |
| 11 | Guanajuato | hw_95_min | 0.999 | 0.984 | 1.015 |
| 11 | Guanajuato | hw_99_2 | 1.082 | 1.039 | 1.127 |
| 11 | Guanajuato | hw_99 | 1.072 | 1.038 | 1.108 |
| 11 | Guanajuato | hw_99_lag1 | 1.079 | 1.044 | 1.115 |
| 11 | Guanajuato | hw_99_lag2 | 1.070 | 1.036 | 1.106 |
| 12 | Guerrero | hw_95 | 1.067 | 1.045 | 1.090 |
| 12 | Guerrero | hw_99 | 1.089 | 1.047 | 1.133 |
| 12 | Guerrero | hw_35abs | 1.070 | 1.050 | 1.090 |
| 12 | Guerrero | hw_99_min | 1.081 | 1.038 | 1.125 |
| 12 | Guerrero | hw_95_min | 1.040 | 1.019 | 1.061 |
| 12 | Guerrero | hw_30abs | 1.017 | 1.000 | 1.035 |
| 12 | Guerrero | hw_99 | 1.089 | 1.047 | 1.133 |
| 12 | Guerrero | hw_99_lag1 | 1.044 | 1.003 | 1.087 |
| 12 | Guerrero | hw_99_lag2 | 1.032 | 0.991 | 1.074 |
| 12 | Guerrero | hw_99_2 | 1.073 | 1.010 | 1.139 |
| 13 | Hidalgo | hw_99 | 1.063 | 1.014 | 1.114 |
| 13 | Hidalgo | hw_35abs | 1.097 | 1.047 | 1.150 |
| 13 | Hidalgo | hw_99_lag1 | 1.006 | 0.959 | 1.056 |
| 13 | Hidalgo | hw_99_lag2 | 1.016 | 0.968 | 1.066 |
| 13 | Hidalgo | hw_99_2 | 0.991 | 0.928 | 1.057 |
| 13 | Hidalgo | hw_30abs | 1.004 | 0.981 | 1.027 |
| 13 | Hidalgo | hw_95 | 1.053 | 1.029 | 1.078 |
| 13 | Hidalgo | hw_99_min | 1.029 | 0.986 | 1.074 |
| 13 | Hidalgo | hw_95_min | 1.013 | 0.990 | 1.036 |
| 13 | Hidalgo | hw_99 | 1.063 | 1.014 | 1.114 |
| 14 | Jalisco | hw_99 | 1.077 | 1.050 | 1.106 |
| 14 | Jalisco | hw_99_min | 1.027 | 1.002 | 1.053 |
| 14 | Jalisco | hw_95_min | 1.012 | 1.000 | 1.025 |
| 14 | Jalisco | hw_30abs | 1.030 | 1.021 | 1.039 |
| 14 | Jalisco | hw_95 | 1.059 | 1.045 | 1.074 |
| 14 | Jalisco | hw_99 | 1.077 | 1.050 | 1.106 |
| 14 | Jalisco | hw_35abs | 1.057 | 1.033 | 1.081 |
| 14 | Jalisco | hw_99_lag1 | 1.089 | 1.061 | 1.117 |
| 14 | Jalisco | hw_99_lag2 | 1.068 | 1.040 | 1.096 |
| 14 | Jalisco | hw_99_2 | 1.084 | 1.047 | 1.122 |
| 15 | Mexico | hw_99_min | 1.006 | 0.987 | 1.026 |
| 15 | Mexico | hw_95_min | 0.993 | 0.983 | 1.004 |
| 15 | Mexico | hw_30abs | 1.039 | 1.023 | 1.055 |
| 15 | Mexico | hw_35abs | 1.083 | 0.967 | 1.213 |
| 15 | Mexico | hw_99_lag1 | 1.023 | 1.001 | 1.044 |
| 15 | Mexico | hw_99_lag2 | 1.004 | 0.983 | 1.026 |
| 15 | Mexico | hw_99_2 | 1.017 | 0.990 | 1.045 |
| 15 | Mexico | hw_99 | 1.042 | 1.021 | 1.064 |
| 15 | Mexico | hw_95 | 1.022 | 1.011 | 1.034 |
| 15 | Mexico | hw_99 | 1.042 | 1.021 | 1.064 |
| 16 | Michoacan de Ocampo | hw_99 | 1.093 | 1.056 | 1.132 |
| 16 | Michoacan de Ocampo | hw_99_2 | 1.099 | 1.051 | 1.149 |
| 16 | Michoacan de Ocampo | hw_30abs | 1.026 | 1.012 | 1.040 |
| 16 | Michoacan de Ocampo | hw_95 | 1.051 | 1.032 | 1.071 |
| 16 | Michoacan de Ocampo | hw_99_min | 1.032 | 0.999 | 1.066 |
| 16 | Michoacan de Ocampo | hw_95_min | 0.995 | 0.978 | 1.012 |
| 16 | Michoacan de Ocampo | hw_99 | 1.093 | 1.056 | 1.132 |
| 16 | Michoacan de Ocampo | hw_35abs | 1.029 | 1.001 | 1.058 |
| 16 | Michoacan de Ocampo | hw_99_lag1 | 1.086 | 1.048 | 1.124 |
| 16 | Michoacan de Ocampo | hw_99_lag2 | 1.066 | 1.030 | 1.105 |
| 17 | Morelos | hw_99 | 1.100 | 1.037 | 1.166 |
| 17 | Morelos | hw_95 | 1.074 | 1.043 | 1.106 |
| 17 | Morelos | hw_99_min | 1.030 | 0.976 | 1.087 |
| 17 | Morelos | hw_95_min | 1.017 | 0.991 | 1.044 |
| 17 | Morelos | hw_30abs | 1.031 | 1.014 | 1.048 |
| 17 | Morelos | hw_99 | 1.100 | 1.037 | 1.166 |
| 17 | Morelos | hw_35abs | 1.097 | 1.048 | 1.148 |
| 17 | Morelos | hw_99_lag1 | 1.096 | 1.034 | 1.163 |
| 17 | Morelos | hw_99_lag2 | 1.049 | 0.988 | 1.114 |
| 17 | Morelos | hw_99_2 | 1.123 | 1.041 | 1.211 |
| 18 | Nayarit | hw_35abs | 1.068 | 1.029 | 1.109 |
| 18 | Nayarit | hw_99_lag1 | 1.025 | 0.955 | 1.100 |
| 18 | Nayarit | hw_99_lag2 | 0.998 | 0.930 | 1.071 |
| 18 | Nayarit | hw_99_2 | 0.989 | 0.878 | 1.114 |
| 18 | Nayarit | hw_99 | 1.052 | 0.980 | 1.128 |
| 18 | Nayarit | hw_95 | 1.068 | 1.030 | 1.107 |
| 18 | Nayarit | hw_99_min | 1.018 | 0.953 | 1.089 |
| 18 | Nayarit | hw_95_min | 1.011 | 0.974 | 1.048 |
| 18 | Nayarit | hw_30abs | 1.038 | 1.017 | 1.061 |
| 18 | Nayarit | hw_99 | 1.052 | 0.980 | 1.128 |
| 19 | Nuevo Leon | hw_99 | 1.114 | 1.078 | 1.151 |
| 19 | Nuevo Leon | hw_95 | 1.074 | 1.056 | 1.092 |
| 19 | Nuevo Leon | hw_99_min | 1.057 | 1.025 | 1.090 |
| 19 | Nuevo Leon | hw_95_min | 1.028 | 1.012 | 1.045 |
| 19 | Nuevo Leon | hw_99 | 1.114 | 1.078 | 1.151 |
| 19 | Nuevo Leon | hw_35abs | 1.054 | 1.042 | 1.065 |
| 19 | Nuevo Leon | hw_99_lag1 | 1.124 | 1.088 | 1.162 |
| 19 | Nuevo Leon | hw_99_lag2 | 1.127 | 1.091 | 1.164 |
| 19 | Nuevo Leon | hw_30abs | 1.019 | 1.009 | 1.029 |
| 19 | Nuevo Leon | hw_99_2 | 1.182 | 1.129 | 1.237 |
| 20 | Oaxaca | hw_99_2 | 1.090 | 1.035 | 1.149 |
| 20 | Oaxaca | hw_99 | 1.054 | 1.015 | 1.095 |
| 20 | Oaxaca | hw_95 | 1.056 | 1.036 | 1.076 |
| 20 | Oaxaca | hw_99_min | 1.037 | 1.001 | 1.075 |
| 20 | Oaxaca | hw_95_min | 1.023 | 1.004 | 1.041 |
| 20 | Oaxaca | hw_30abs | 1.014 | 1.002 | 1.027 |
| 20 | Oaxaca | hw_35abs | 1.075 | 1.057 | 1.094 |
| 20 | Oaxaca | hw_99_lag1 | 1.061 | 1.022 | 1.102 |
| 20 | Oaxaca | hw_99_lag2 | 1.046 | 1.007 | 1.087 |
| 20 | Oaxaca | hw_99 | 1.054 | 1.015 | 1.095 |
| 21 | Puebla | hw_99 | 1.013 | 0.984 | 1.043 |
| 21 | Puebla | hw_95 | 1.041 | 1.026 | 1.057 |
| 21 | Puebla | hw_99_min | 0.997 | 0.969 | 1.025 |
| 21 | Puebla | hw_95_min | 1.009 | 0.995 | 1.023 |
| 21 | Puebla | hw_30abs | 1.029 | 1.015 | 1.042 |
| 21 | Puebla | hw_35abs | 1.107 | 1.065 | 1.151 |
| 21 | Puebla | hw_99_lag1 | 1.026 | 0.997 | 1.057 |
| 21 | Puebla | hw_99_lag2 | 0.987 | 0.958 | 1.016 |
| 21 | Puebla | hw_99_2 | 1.034 | 0.992 | 1.078 |
| 21 | Puebla | hw_99 | 1.013 | 0.984 | 1.043 |
| 22 | Queretaro | hw_95 | 1.040 | 1.009 | 1.072 |
| 22 | Queretaro | hw_99_min | 1.022 | 0.968 | 1.079 |
| 22 | Queretaro | hw_95_min | 0.991 | 0.963 | 1.019 |
| 22 | Queretaro | hw_99 | 1.070 | 1.011 | 1.134 |
| 22 | Queretaro | hw_99 | 1.070 | 1.011 | 1.134 |
| 22 | Queretaro | hw_35abs | 1.076 | 0.953 | 1.216 |
| 22 | Queretaro | hw_99_lag1 | 1.060 | 1.000 | 1.123 |
| 22 | Queretaro | hw_99_lag2 | 1.079 | 1.019 | 1.142 |
| 22 | Queretaro | hw_99_2 | 1.078 | 1.004 | 1.158 |
| 22 | Queretaro | hw_30abs | 1.025 | 1.002 | 1.049 |
| 23 | Quintana Roo | hw_95 | 1.021 | 0.984 | 1.060 |
| 23 | Quintana Roo | hw_99_min | 1.022 | 0.953 | 1.096 |
| 23 | Quintana Roo | hw_95_min | 1.026 | 0.991 | 1.062 |
| 23 | Quintana Roo | hw_99 | 1.036 | 0.957 | 1.121 |
| 23 | Quintana Roo | hw_35abs | 1.004 | 0.974 | 1.035 |
| 23 | Quintana Roo | hw_99_lag1 | 0.958 | 0.886 | 1.036 |
| 23 | Quintana Roo | hw_99_lag2 | 1.032 | 0.955 | 1.115 |
| 23 | Quintana Roo | hw_30abs | 1.029 | 1.004 | 1.054 |
| 23 | Quintana Roo | hw_99 | 1.036 | 0.957 | 1.121 |
| 23 | Quintana Roo | hw_99_2 | 0.959 | 0.839 | 1.096 |
| 24 | San Luis Potosi | hw_99_min | 1.077 | 1.031 | 1.125 |
| 24 | San Luis Potosi | hw_95_min | 1.049 | 1.025 | 1.073 |
| 24 | San Luis Potosi | hw_30abs | 1.051 | 1.036 | 1.066 |
| 24 | San Luis Potosi | hw_99_lag1 | 1.142 | 1.090 | 1.196 |
| 24 | San Luis Potosi | hw_99_lag2 | 1.040 | 0.992 | 1.091 |
| 24 | San Luis Potosi | hw_99_2 | 1.194 | 1.122 | 1.271 |
| 24 | San Luis Potosi | hw_35abs | 1.053 | 1.030 | 1.077 |
| 24 | San Luis Potosi | hw_99 | 1.168 | 1.116 | 1.222 |
| 24 | San Luis Potosi | hw_95 | 1.078 | 1.054 | 1.103 |
| 24 | San Luis Potosi | hw_99 | 1.168 | 1.116 | 1.222 |
| 25 | Sinaloa | hw_99 | 1.094 | 1.045 | 1.145 |
| 25 | Sinaloa | hw_95 | 1.056 | 1.033 | 1.080 |
| 25 | Sinaloa | hw_99_min | 1.081 | 1.036 | 1.127 |
| 25 | Sinaloa | hw_95_min | 1.034 | 1.012 | 1.057 |
| 25 | Sinaloa | hw_99_2 | 1.091 | 1.016 | 1.173 |
| 25 | Sinaloa | hw_30abs | 1.008 | 0.993 | 1.022 |
| 25 | Sinaloa | hw_35abs | 1.026 | 1.012 | 1.040 |
| 25 | Sinaloa | hw_99_lag1 | 1.031 | 0.985 | 1.079 |
| 25 | Sinaloa | hw_99_lag2 | 1.083 | 1.034 | 1.133 |
| 25 | Sinaloa | hw_99 | 1.094 | 1.045 | 1.145 |
| 26 | Sonora | hw_95 | 1.073 | 1.051 | 1.096 |
| 26 | Sonora | hw_99_min | 1.094 | 1.050 | 1.140 |
| 26 | Sonora | hw_95_min | 1.054 | 1.032 | 1.077 |
| 26 | Sonora | hw_99 | 1.125 | 1.080 | 1.172 |
| 26 | Sonora | hw_35abs | 1.029 | 1.014 | 1.045 |
| 26 | Sonora | hw_99_lag1 | 1.111 | 1.066 | 1.158 |
| 26 | Sonora | hw_99_lag2 | 1.053 | 1.010 | 1.098 |
| 26 | Sonora | hw_30abs | 1.011 | 0.997 | 1.025 |
| 26 | Sonora | hw_99 | 1.125 | 1.080 | 1.172 |
| 26 | Sonora | hw_99_2 | 1.164 | 1.086 | 1.248 |
| 27 | Tabasco | hw_35abs | 1.071 | 1.056 | 1.086 |
| 27 | Tabasco | hw_99_lag1 | 1.207 | 1.147 | 1.269 |
| 27 | Tabasco | hw_99_lag2 | 1.155 | 1.097 | 1.215 |
| 27 | Tabasco | hw_99 | 1.235 | 1.174 | 1.298 |
| 27 | Tabasco | hw_99 | 1.235 | 1.174 | 1.298 |
| 27 | Tabasco | hw_95 | 1.137 | 1.110 | 1.165 |
| 27 | Tabasco | hw_99_min | 1.110 | 1.061 | 1.161 |
| 27 | Tabasco | hw_99_2 | 1.273 | 1.192 | 1.360 |
| 27 | Tabasco | hw_30abs | 1.019 | 1.003 | 1.036 |
| 27 | Tabasco | hw_95_min | 1.077 | 1.052 | 1.102 |
| 28 | Tamaulipas | hw_95 | 1.058 | 1.037 | 1.079 |
| 28 | Tamaulipas | hw_99_min | 1.059 | 1.020 | 1.099 |
| 28 | Tamaulipas | hw_99 | 1.124 | 1.083 | 1.167 |
| 28 | Tamaulipas | hw_99 | 1.124 | 1.083 | 1.167 |
| 28 | Tamaulipas | hw_35abs | 1.036 | 1.021 | 1.050 |
| 28 | Tamaulipas | hw_99_lag1 | 1.102 | 1.061 | 1.144 |
| 28 | Tamaulipas | hw_95_min | 1.044 | 1.024 | 1.064 |
| 28 | Tamaulipas | hw_30abs | 1.013 | 1.001 | 1.026 |
| 28 | Tamaulipas | hw_99_lag2 | 1.054 | 1.015 | 1.095 |
| 28 | Tamaulipas | hw_99_2 | 1.154 | 1.090 | 1.221 |
| 29 | Tlaxcala | hw_99_min | 0.992 | 0.928 | 1.060 |
| 29 | Tlaxcala | hw_95_min | 0.978 | 0.946 | 1.012 |
| 29 | Tlaxcala | hw_99_lag1 | 1.000 | 0.930 | 1.077 |
| 29 | Tlaxcala | hw_99_lag2 | 1.000 | 0.929 | 1.077 |
| 29 | Tlaxcala | hw_30abs | 1.011 | 0.953 | 1.074 |
| 29 | Tlaxcala | hw_35abs | 8.000 | 0.725 | 88.225 |
| 29 | Tlaxcala | hw_99 | 1.016 | 0.945 | 1.093 |
| 29 | Tlaxcala | hw_95 | 1.010 | 0.973 | 1.049 |
| 29 | Tlaxcala | hw_99 | 1.016 | 0.945 | 1.093 |
| 29 | Tlaxcala | hw_99_2 | 1.043 | 0.942 | 1.155 |
| 30 | Veracruz de Ignacio de la Llave | hw_95 | 1.101 | 1.088 | 1.113 |
| 30 | Veracruz de Ignacio de la Llave | hw_99_min | 1.123 | 1.098 | 1.148 |
| 30 | Veracruz de Ignacio de la Llave | hw_99 | 1.143 | 1.117 | 1.170 |
| 30 | Veracruz de Ignacio de la Llave | hw_99 | 1.143 | 1.117 | 1.170 |
| 30 | Veracruz de Ignacio de la Llave | hw_30abs | 1.042 | 1.034 | 1.050 |
| 30 | Veracruz de Ignacio de la Llave | hw_35abs | 1.101 | 1.091 | 1.112 |
| 30 | Veracruz de Ignacio de la Llave | hw_99_lag1 | 1.132 | 1.106 | 1.159 |
| 30 | Veracruz de Ignacio de la Llave | hw_95_min | 1.069 | 1.056 | 1.082 |
| 30 | Veracruz de Ignacio de la Llave | hw_99_2 | 1.192 | 1.153 | 1.232 |
| 30 | Veracruz de Ignacio de la Llave | hw_99_lag2 | 1.086 | 1.061 | 1.112 |
| 31 | Yucatan | hw_35abs | 1.071 | 1.056 | 1.087 |
| 31 | Yucatan | hw_99_lag1 | 1.136 | 1.083 | 1.191 |
| 31 | Yucatan | hw_99 | 1.155 | 1.102 | 1.210 |
| 31 | Yucatan | hw_99 | 1.155 | 1.102 | 1.210 |
| 31 | Yucatan | hw_95 | 1.119 | 1.092 | 1.146 |
| 31 | Yucatan | hw_99_min | 1.062 | 1.016 | 1.110 |
| 31 | Yucatan | hw_95_min | 1.054 | 1.031 | 1.079 |
| 31 | Yucatan | hw_30abs | 1.004 | 0.990 | 1.019 |
| 31 | Yucatan | hw_99_lag2 | 1.096 | 1.045 | 1.150 |
| 31 | Yucatan | hw_99_2 | 1.166 | 1.095 | 1.242 |
| 32 | Zacatecas | hw_99 | 1.014 | 0.955 | 1.077 |
| 32 | Zacatecas | hw_95 | 1.076 | 1.044 | 1.110 |
| 32 | Zacatecas | hw_99 | 1.014 | 0.955 | 1.077 |
| 32 | Zacatecas | hw_35abs | 1.222 | 1.119 | 1.335 |
| 32 | Zacatecas | hw_99_min | 1.031 | 0.971 | 1.095 |
| 32 | Zacatecas | hw_95_min | 1.029 | 0.999 | 1.061 |
| 32 | Zacatecas | hw_30abs | 1.031 | 1.005 | 1.057 |
| 32 | Zacatecas | hw_99_lag1 | 1.041 | 0.981 | 1.104 |
| 32 | Zacatecas | hw_99_lag2 | 1.049 | 0.989 | 1.112 |
| 32 | Zacatecas | hw_99_2 | 1.000 | 0.925 | 1.080 |


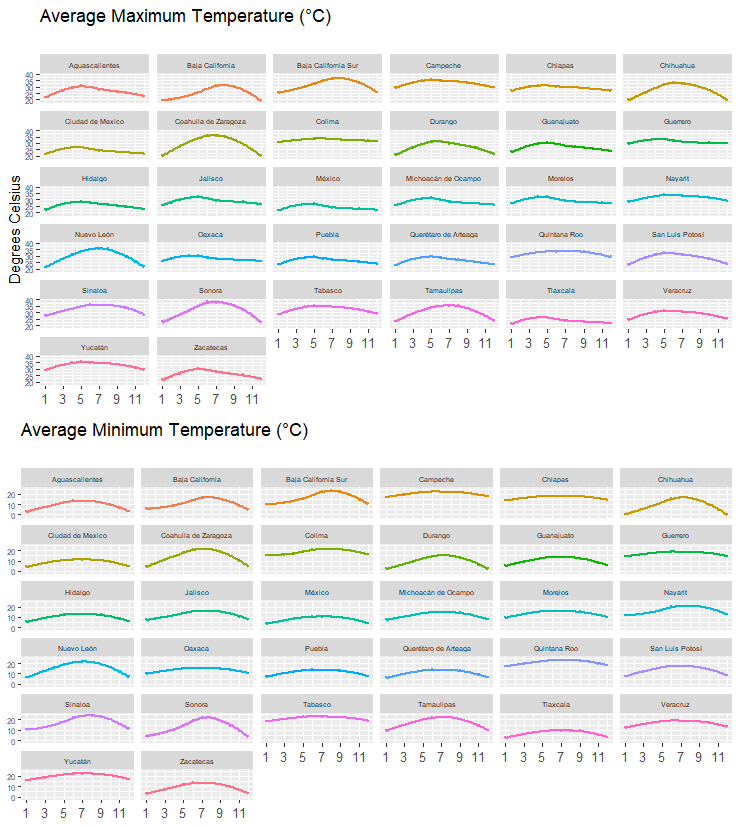


**Supplementary Figure 1:** Average monthly temperature distribution across 32 Mexican states, 1998-2020.

**
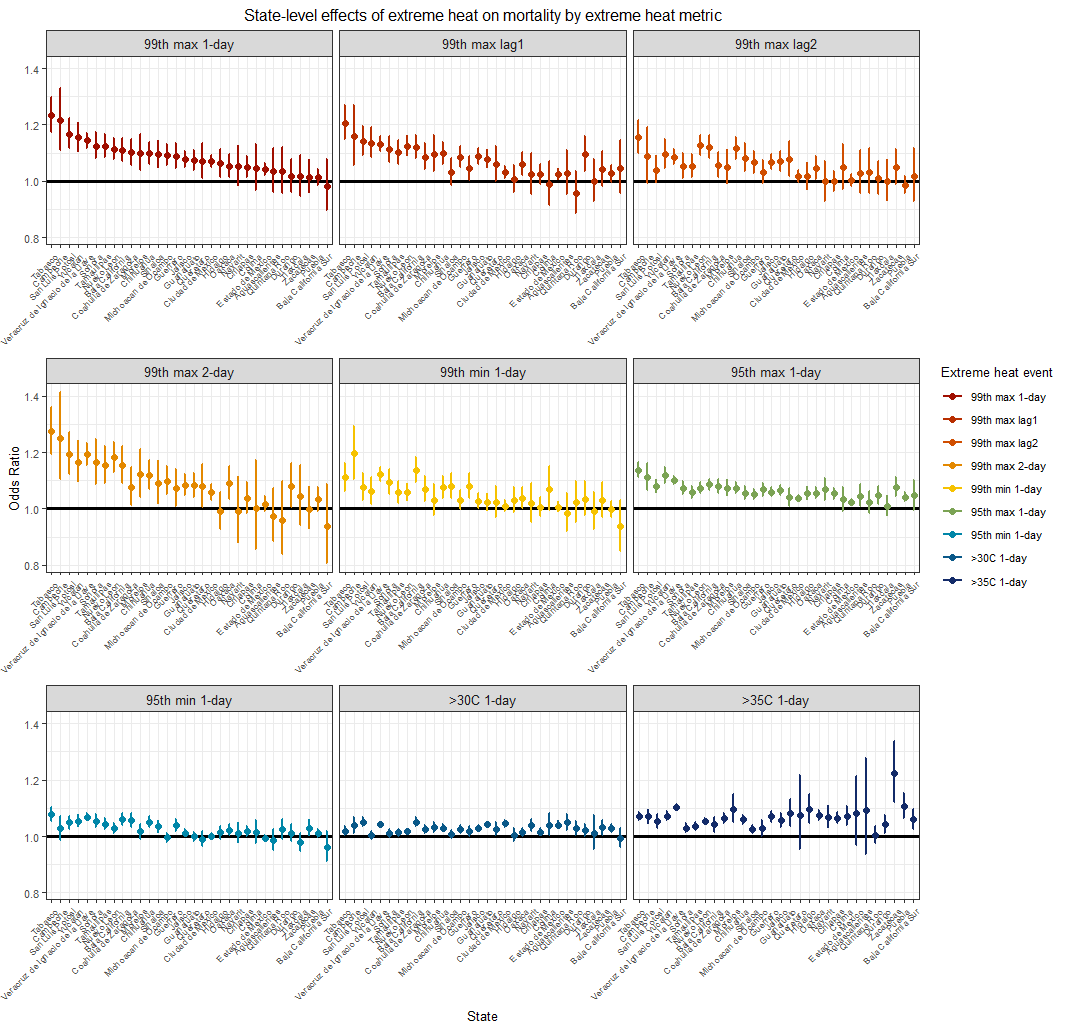
**

**Supplementary Figure 2:** State-level effects of extreme heat on mortality using different extreme heat event metrics, 1998-2019.

**Supplementary Figure 3:** Results of the municipalities with signal-to-noise (SNR) ratio (precise if |SNR| > 2) of the Bayesian resolved estimates of the effect 99^th^ percentile maximum extreme heat events (top left), 95^th^ percentile maximum (top right), 99^th^ percentile minimum (middle right), >95^th^ percentile minimum (middle right), 30°C (bottom left) and >35°C (bottom right) on all-cause mortality across Mexico, 1998-2019.

**Supplementary Figure 4:** Bayesian resolved estimates of the excess relative risk of six measures of extreme heat (99^th^ max, 95^th^ max, 99^th^ min, 95^th^ min, > 30°C, > 35°C).

**Supplementary Figure 5:** Bayesian resolved estimates for ERR (left) and associated signal-noise ratio (SNR) (right) for sensitivity analysis in Mexico including 2020 in the study period for the 99^th^ percentile maximum temperature measure of extreme heat, 1998-2020.

**Supplementary Figure 6:** Sensitivity analysis of municipality-level analysis using 99^th^ maximum temperature extreme heat events using 6 day exclusion criteria (top) and 30 day before and after extreme heat day as control period (bottom), 1998-2019.

**
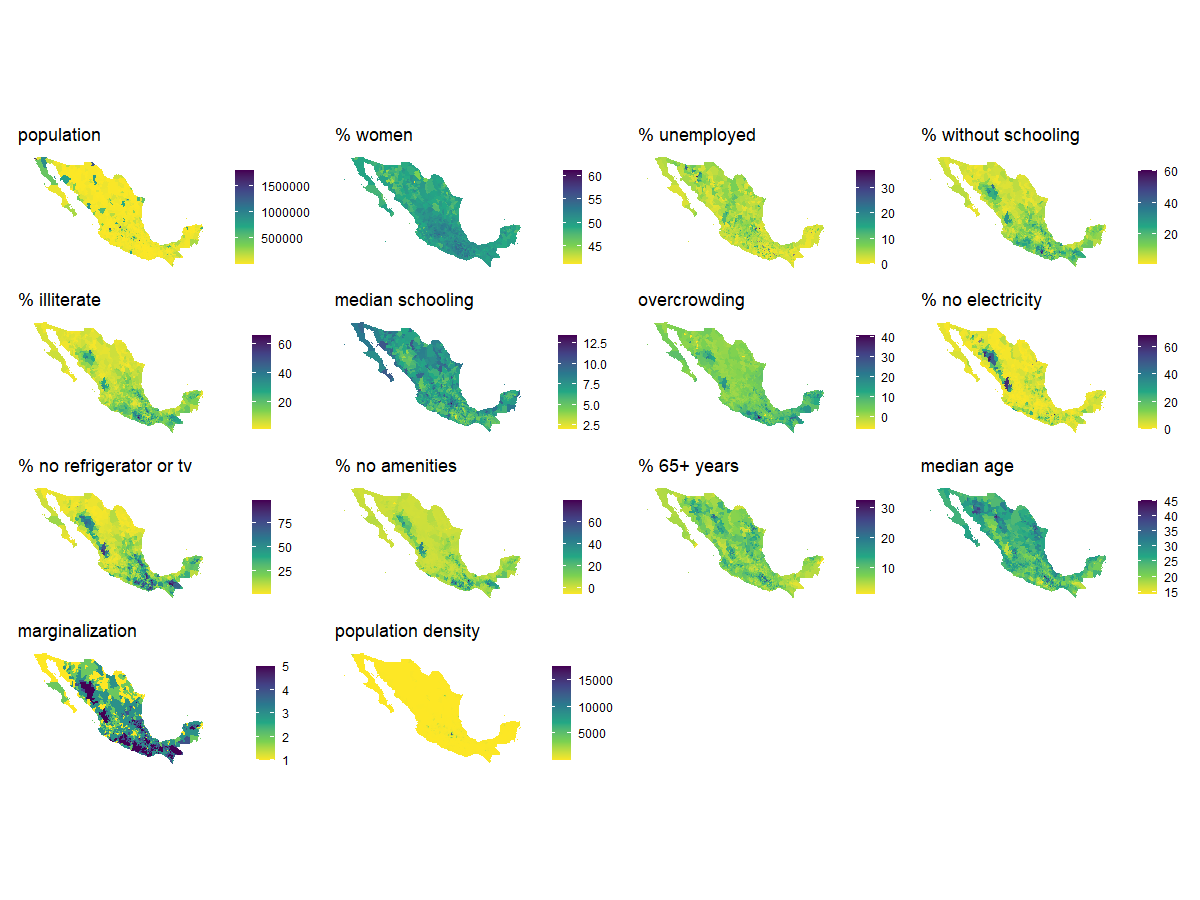
**

**Supplementary Figure 7:** Descriptive maps of socio-demographics at municipality-level considered in meta-regression.

**
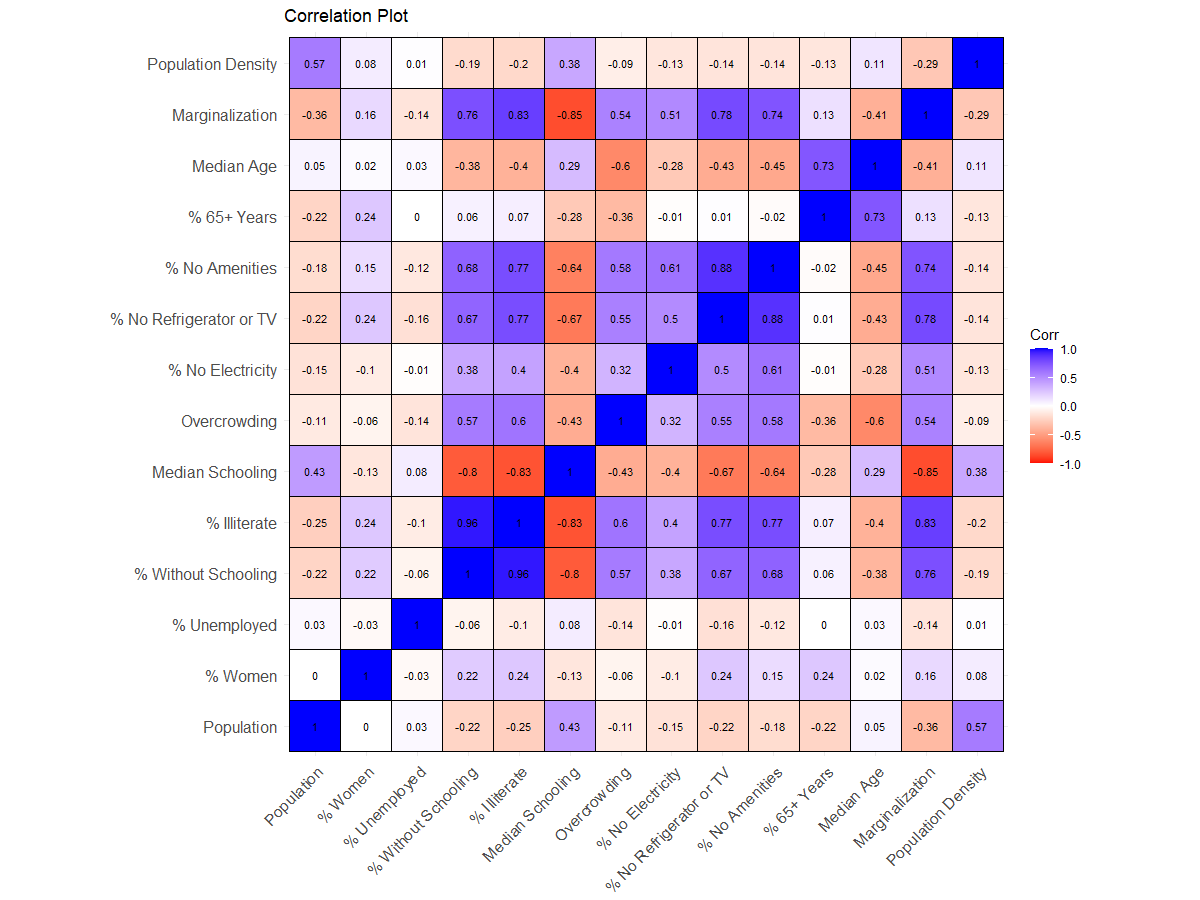
**

**Supplementary Figure 8:** Correlation matrix of socio-demographics considered in meta-regression.


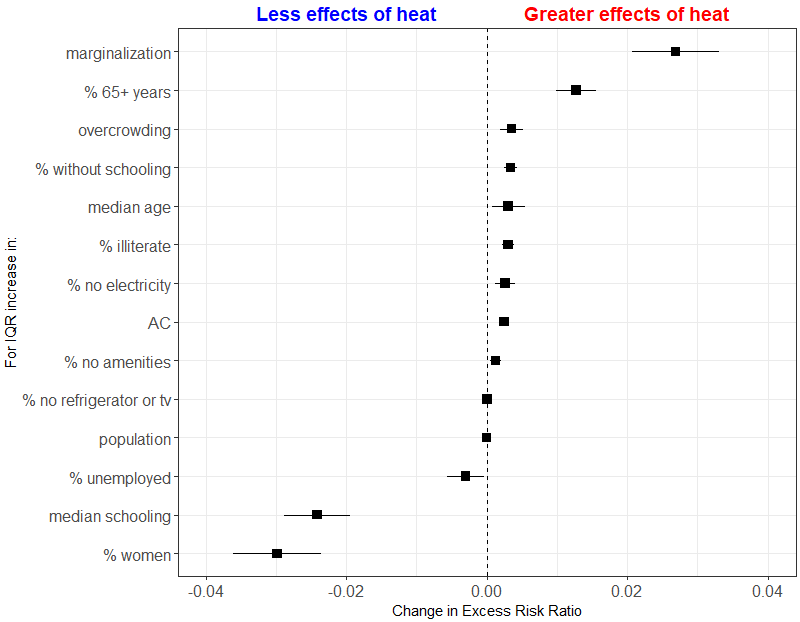

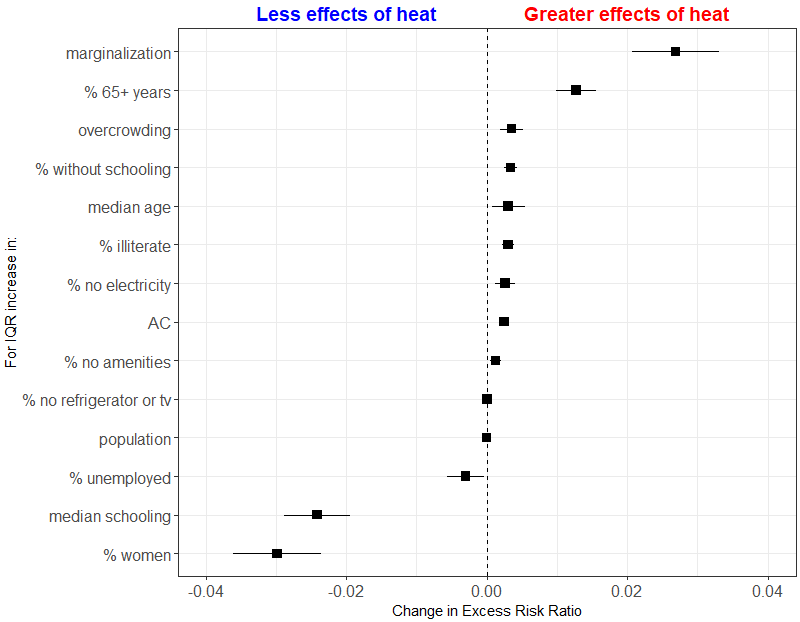

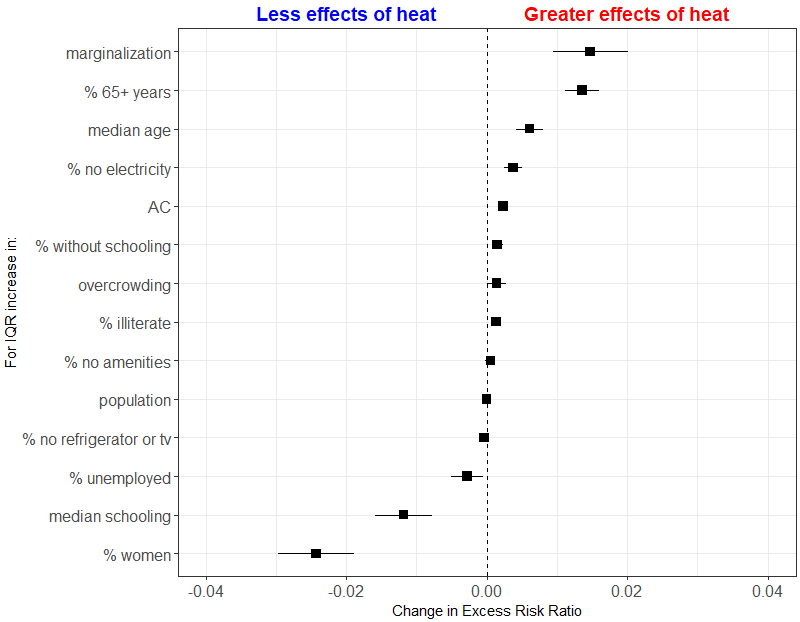

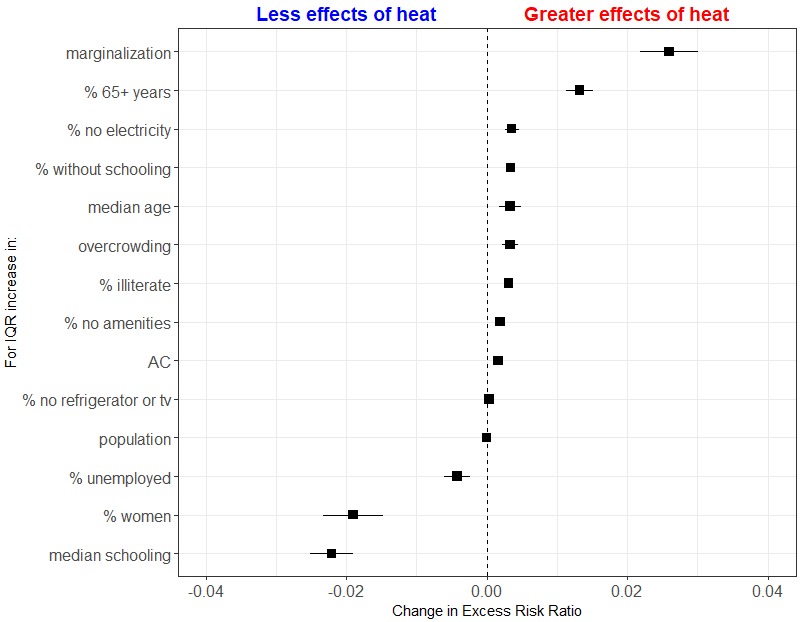

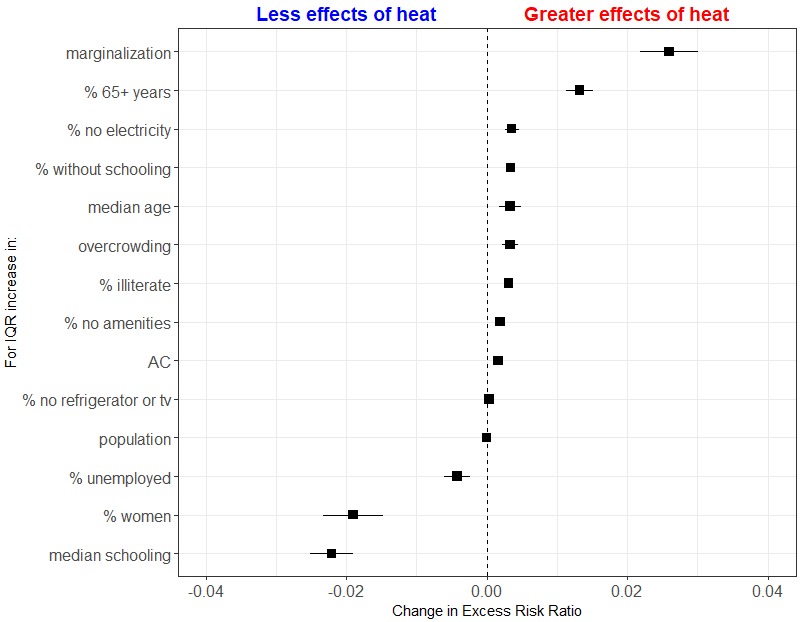

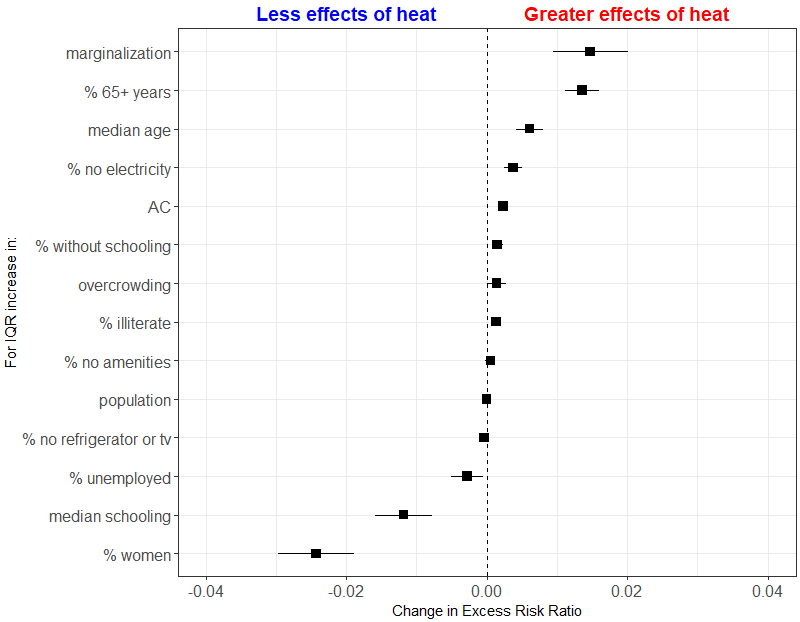

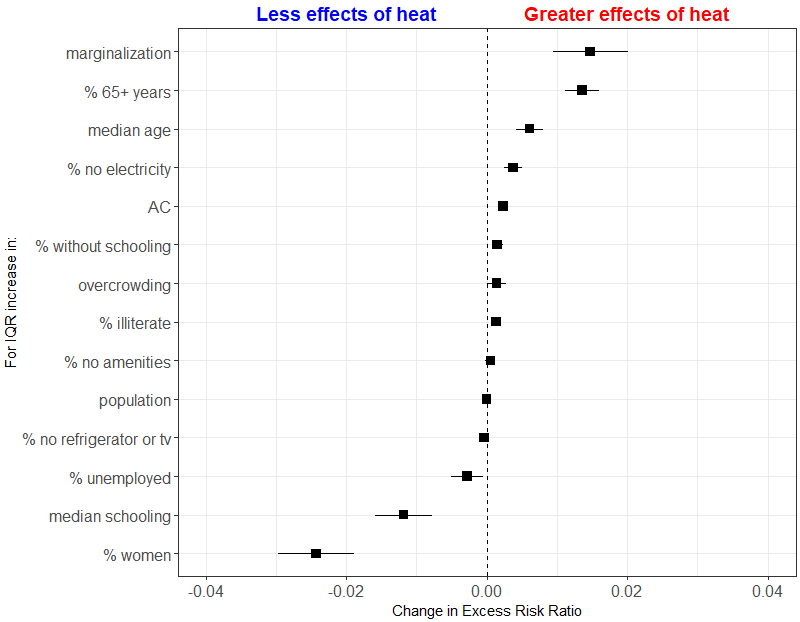

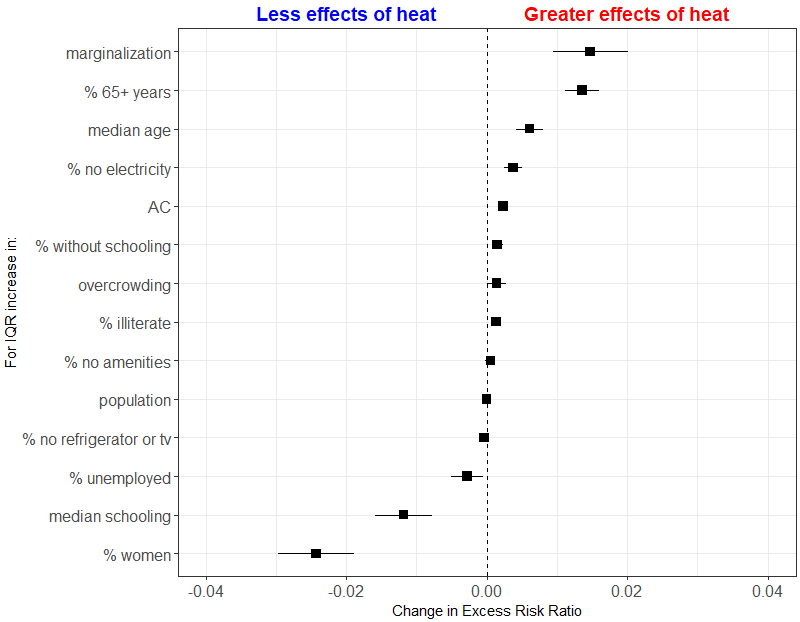

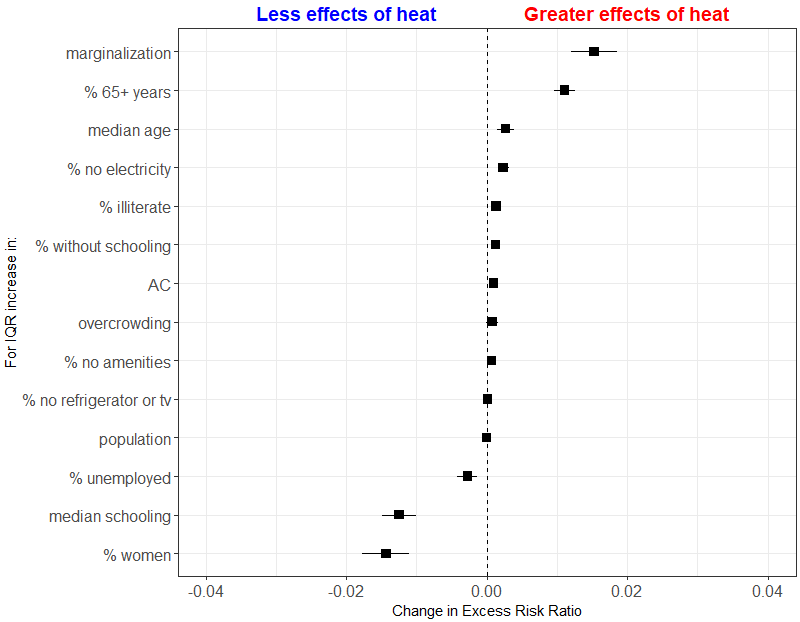

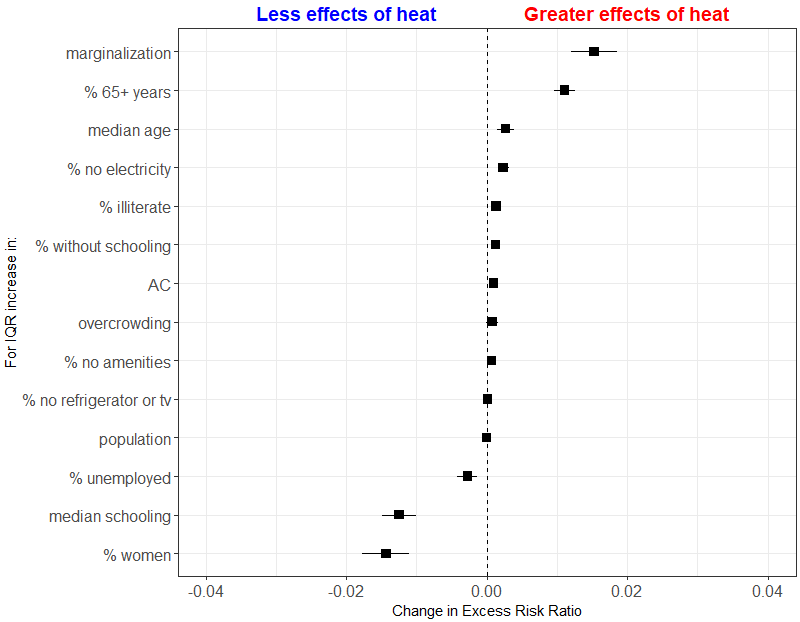

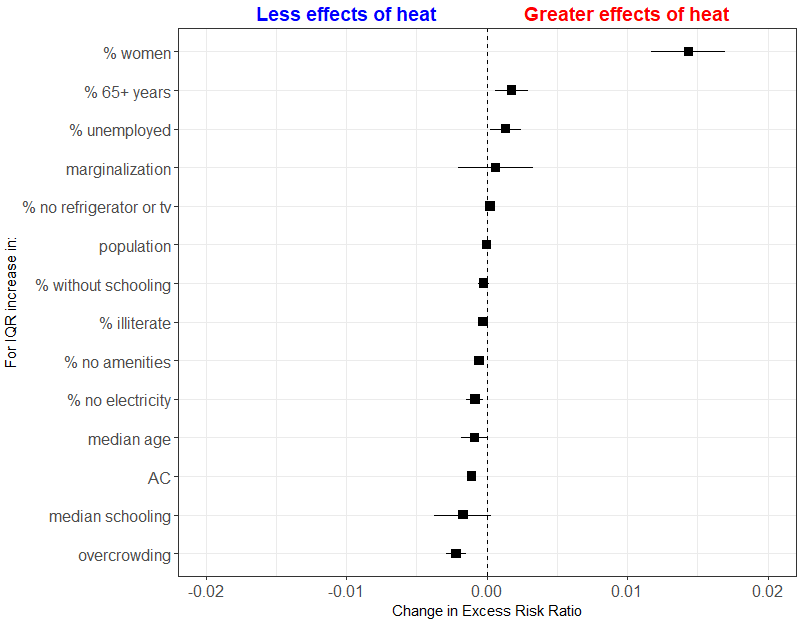

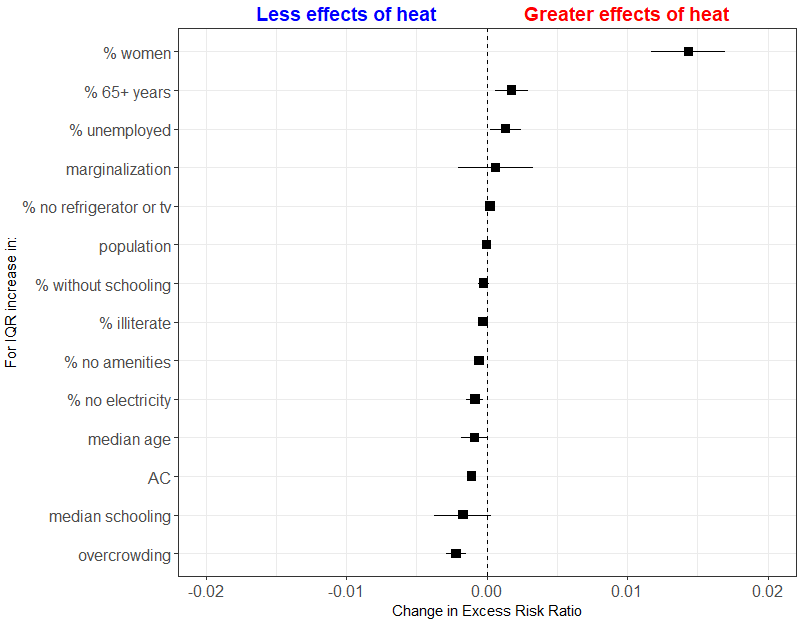

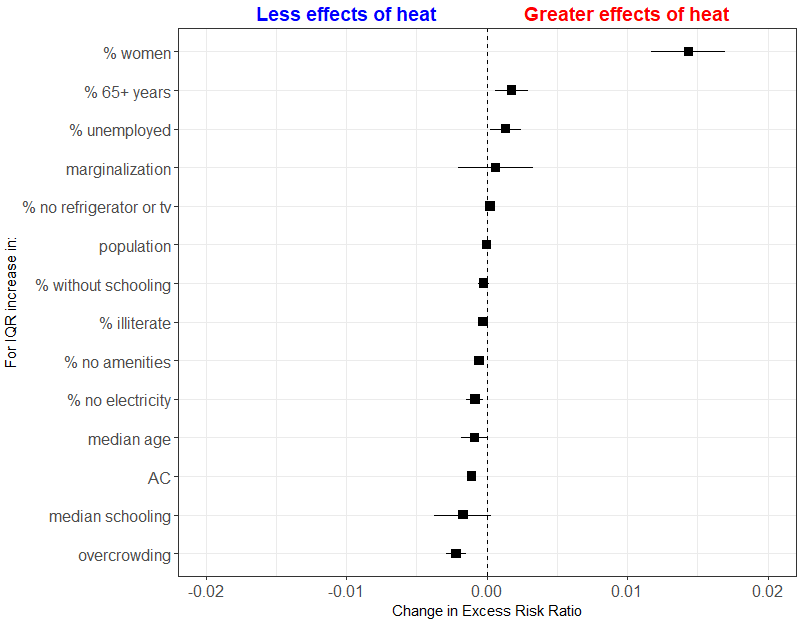

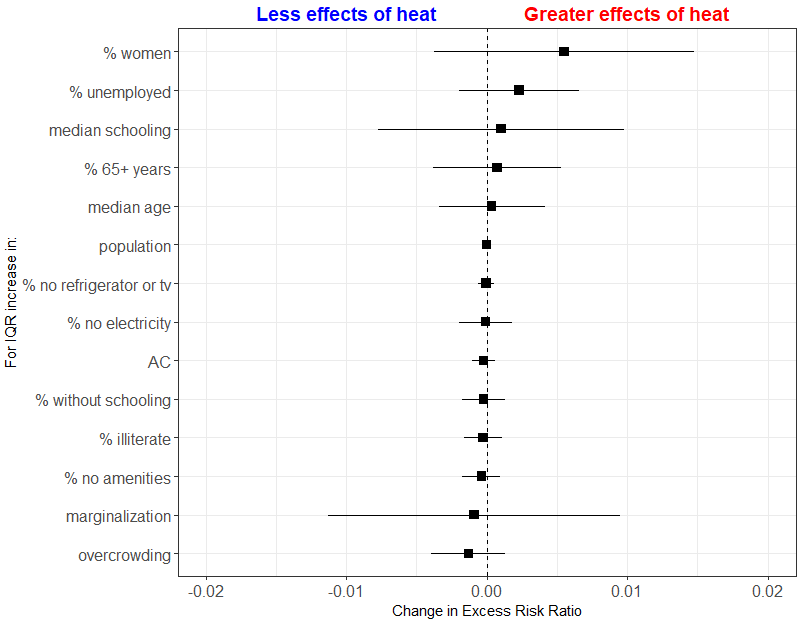

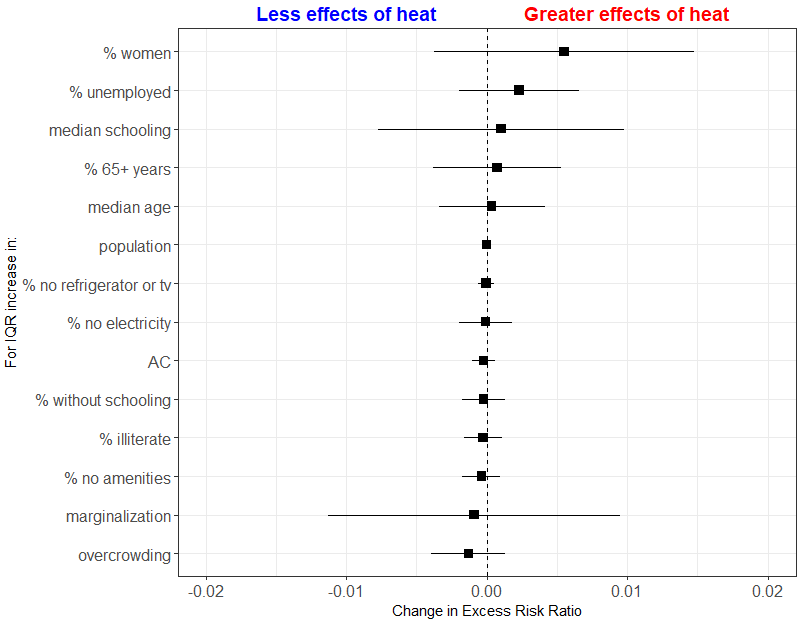

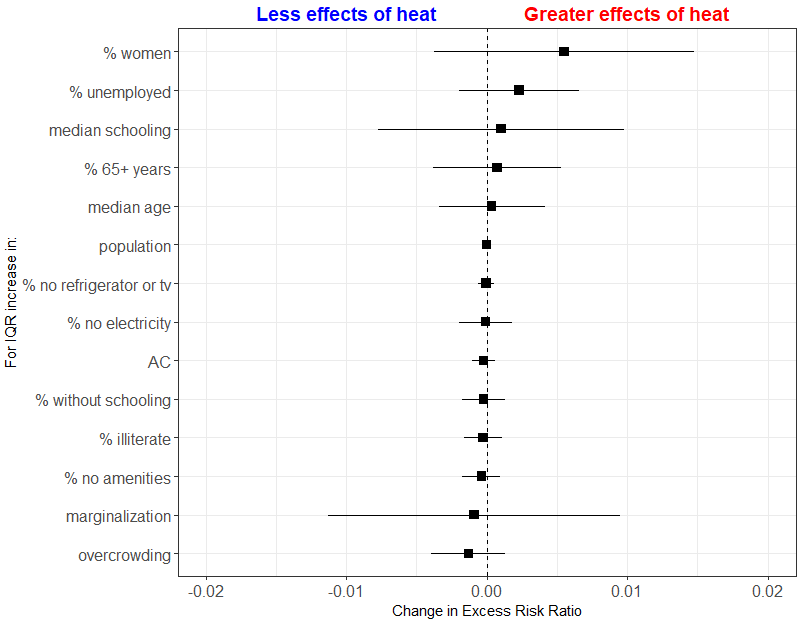


**Supplementary Figure 9:** Results of meta-regression for various metrics of extreme heat 99th percentile maximum extreme heat events (top left), 95th percentile maximum (top right), 99th percentile minimum extreme heat events (middle left), >95th percentile minimum (middle right), 30°C (bottom left) and >35°C (bottom right)**.**
